# The early identification of disease progression in patients with suspected infection presenting to the emergency department: a multi-centre derivation and validation study

**DOI:** 10.1186/s13054-019-2329-5

**Published:** 2019-02-08

**Authors:** Kordo Saeed, Darius Cameron Wilson, Frank Bloos, Philipp Schuetz, Yuri van der Does, Olle Melander, Pierre Hausfater, Jacopo M. Legramante, Yann-Erick Claessens, Deveendra Amin, Mari Rosenqvist, Graham White, Beat Mueller, Maarten Limper, Carlota Clemente Callejo, Antonella Brandi, Marc-Alexis Macchi, Nicholas Cortes, Alexander Kutz, Peter Patka, María Cecilia Yañez, Sergio Bernardini, Nathalie Beau, Matthew Dryden, Eric C. M. van Gorp, Marilena Minieri, Louisa Chan, Pleunie P. M. Rood, Juan Gonzalez del Castillo

**Affiliations:** 1 Department of Microbiology, Hampshire Hospitals NHS Foundation Trust, Winchester and Basingstoke, UK; 2 University of Southampton, School of Medicine, Southampton, UK; 3 B·R·A·H·M·S GmbH, Hennigsdorf, Germany; 4 Department of Anesthesiology and Intensive Care Medicine, Jena University Hospital, Jena, Germany; 5 Center for Sepsis Control & Care (CSCC), Jena University Hospital, Jena, Germany; 6 Division of General and Emergency Medicine, University Department of Medicine, Kantonsspital Aarau, Switzerland; 7 Medical Faculty of the University of Basel, Basel, Switzerland; 8 Department of Emergency Medicine, Erasmus University Medical Center, Rotterdam, Netherlands; 9 Department of Internal Medicine, Skåne University Hospital, Malmö, Sweden; 10 Department of Clinical Sciences Malmö, Lund University, Lund, Sweden; 11 Emergency Department hôpital Pitié-Salpêtrière, Assistance Publique - Hôpitaux de Paris and Sorbonne Universités GRC-14 BIOSFAST and INSERM UMR-S 1166, Paris, France; 12 Emergency Department, Policlinico Tor Vergata, Rome, Italy; 13 Department of Medical Systems, Universita di Tor Vergata, Rome, Italy; 14 Department of Emergency Medicine, Monaco Princess Grace Hospital, Monaco, France; 15 Department of Critical Care, Morton Plant Hospital, 300 Pinellas Street, Clearwater, FL 33756 USA; 16 Infectious Disease Unit, Skåne University Hospital, Malmö, Sweden; 17 Department of Blood Sciences, Hampshire Hospitals NHS Foundation Trust, Winchester and Basingstoke, UK; 18 Department of Rheumatology and Clinical Immunology, University Medical Center, Utrecht University, Utrecht, Netherlands; 19 Emergency Department, Hospital Clínico San Carlos, Madrid, Spain; 20 Gibraltar Health Authority, St Bernard’s Hospital, Gibraltar, Spain; 21 Department of Laboratory Medicine, Policlinico Tor Vergata, Rome, Italy; 22 Department of Experimental Medicine, University of Rome Tor Vergata, Rome, Italy; 23 Rare and Imported Pathogen Laboratories, Public Health England, Porton Down, UK; 24 Department of Internal Medicine, Erasmus University Medical Center, Rotterdam, Netherlands; 25 Department of Viroscience, Erasmus University Medical Center, Rotterdam, Netherlands; 26 Department of accident and emergency, Hampshire Hospitals NHS Foundation Trust, Winchester and Basingstoke, UK; 27 Emergency Department, Instituto de Investigación Sanitaria (IdISSC), Hospital Clínico San Carlos, Madrid, Spain

**Keywords:** MR-proADM, Sepsis, SOFA, qSOFA, Disease progression, Emergency department

## Abstract

**Background:**

There is a lack of validated tools to assess potential disease progression and hospitalisation decisions in patients presenting to the emergency department (ED) with a suspected infection. This study aimed to identify suitable blood biomarkers (MR-proADM, PCT, lactate and CRP) or clinical scores (SIRS, SOFA, qSOFA, NEWS and CRB-65) to fulfil this unmet clinical need.

**Methods:**

An observational derivation patient cohort validated by an independent secondary analysis across nine EDs. Logistic and Cox regression, area under the receiver operating characteristic (AUROC) and Kaplan-Meier curves were used to assess performance. Disease progression was identified using a composite endpoint of 28-day mortality, ICU admission and hospitalisation > 10 days.

**Results:**

One thousand one hundred seventy-five derivation and 896 validation patients were analysed with respective 28-day mortality rates of 7.1% and 5.0%, and hospitalisation rates of 77.9% and 76.2%. MR-proADM showed greatest accuracy in predicting 28-day mortality and hospitalisation requirement across both cohorts. Patient subgroups with high MR-proADM concentrations (≥ 1.54 nmol/L) and low biomarker (PCT < 0.25 ng/mL, lactate < 2.0 mmol/L or CRP < 67 mg/L) or clinical score (SOFA < 2 points, qSOFA < 2 points, NEWS < 4 points or CRB-65 < 2 points) values were characterised by a significantly longer length of hospitalisation (*p* < 0.001), rate of ICU admission (*p* < 0.001), elevated mortality risk (e.g. SOFA, qSOFA and NEWS HR [95%CI], 45.5 [10.0–207.6], 23.4 [11.1–49.3] and 32.6 [9.4–113.6], respectively) and a greater number of disease progression events (*p* < 0.001), compared to similar subgroups with low MR-proADM concentrations (< 1.54 nmol/L). Increased out-patient treatment across both cohorts could be facilitated using a derivation-derived MR-proADM cut-off of < 0.87 nmol/L (15.0% and 16.6%), with decreased readmission rates and no mortalities.

**Conclusions:**

In patients presenting to the ED with a suspected infection, the blood biomarker MR-proADM could most accurately identify the likelihood of further disease progression. Incorporation into an early sepsis management protocol may therefore aid rapid decision-making in order to either initiate, escalate or intensify early treatment strategies, or identify patients suitable for safe out-patient treatment.

**Electronic supplementary material:**

The online version of this article (10.1186/s13054-019-2329-5) contains supplementary material, which is available to authorized users.

## Background

All infections have the potential to manifest into life-threatening conditions, depending on the virulence of the infecting organism and the subsequent pathophysiological host response [1]. An early diagnosis and assessment of infection severity is therefore crucial in order to initiate appropriate therapeutic strategies.

Recent changes to the definition and diagnostic criteria used to identify sepsis have resulted in an emphasis on the identification of a dysregulated host response and the presence of life-threatening organ dysfunction [2, 3]. The use of the Sequential Organ Failure Assessment (SOFA) score as part of the clinical criteria to identify and characterise sepsis [2], rather than an emphasis on the non-specific systemic inflammatory response syndrome (SIRS) [4], has proven controversial due to the complex nature of the score. The alternative quick SOFA (qSOFA) score to screen infected patients likely to have a poor outcome has also been reported to have significant sensitivity and kinetical limitations [1, 5–8]. In both cases, a focus on the identification of high severity patients may lead to either a delayed therapeutic response or inappropriate discharge decisions in those with initially low severities but a high potential for disease progression [9, 10]. Such patients at risk of this transitional status have previously been described as “pre-septic” [10]. Conversely, the unnecessary hospitalisation of patients with uncomplicated infections who are at no further risk of disease progression can lead to an additional increase in clinical workload and financial burden. Thus, a more accurate assessment of the pathophysiological host response to infection, and the potential for further disease development, is essential [11, 12].

The use of biological markers which have a high sensitivity for assessing disease severity and are significantly increased during the initial stages of sepsis development may therefore be of significant clinical interest in facilitating early therapeutic decisions [13]. Biomarkers such as procalcitonin (PCT) and C-reactive protein (CRP) are already well established in the field of infectiology [14, 15], whilst elevated lactate levels can reflect significant infection-related cellular dysfunction despite being increased due to other pathophysiological abnormalities [2]. Conversely, the clinical utility of novel biomarkers such as mid-regional proadrenomedullin (MR-proADM) remains less clear. Recent studies have shown MR-proADM concentrations to be rapidly induced in response to LPS stimulation [16] and invasive fungal infections [17], as well as in the initial stages of sepsis development [18] and progression towards sepsis-related multiple organ failure [19, 20]. Thus, MR-proADM may be of significant clinical relevance in settings such as the ED where an early assessment of the potential for further disease progression is vital.

This study therefore aimed to investigate the performance of each biomarker (MR-proADM, PCT, lactate and CRP) and clinical score (SIRS, SOFA, qSOFA, NEWS and CRB-65) in patients presenting to the emergency department with a suspected infection in order to identify (i) those with an increased risk of further disease progression and mortality, and (ii) patients with uncomplicated infections where out-patient treatment may be most appropriate.

## Methods

### Study design and ethical approval

This study analysed and compared results from two patient cohorts. The derivation cohort consisted of patients prospectively enrolled after presenting to the EDs of five hospitals in England, France, Italy, Sweden and Spain between August 2016 and July 2017, with further patients added from a subgroup of a previously published cohort from the Netherlands [21]. The validation cohort consisted of a retrospective subgroup analysis of patients presenting to the EDs of three hospitals in France, Switzerland and the USA [22]. Both cohorts were enrolled in accordance with the Helsinki Declaration. Ethical approval was granted from the relevant boards or governance bodies of each participating hospital, where appropriate, and informed consent obtained from all patients or next of kin. The manuscript was drafted according to the Standards for the Reporting of Diagnostic accuracy studies STARD criteria [23].

### Inclusion and exclusion criteria

Adult patients (≥ 18 years) were enrolled based on a clinical suspicion of infection which could be made according to main presenting symptoms, vital signs, blood culture request or laboratory findings obtained during ED assessment. Exclusion criteria included non-adult patients, pregnancy or refusal to participate. Inclusion and exclusion criteria were similar between the derivation and validation cohorts. An initial blood draw was prospectively taken as part of the routine ED assessment across all sites, and surplus samples stored at − 80 °C for subsequent biomarker measurements.

### Study endpoints and analytical aims

Study endpoints and analytical aims were defined as follows: *28-day mortality*: all-cause mortality within 28 days following enrolment. *Hospitalisation*: hospital admission with a subsequent stay of > 24 h. *Out-patients*: patients presenting to and discharged from the ED on the same day. *Intensive Care Unit (ICU) admission*: all-cause ICU admission within 28 days following enrolment. *Uncomplicated infections*: composite end-point comprising of an absence of 28-day mortality and ICU admission, and a total hospitalisation of ≤ 10 days. *Disease progression*: composite end-point comprising of 28-day mortality, ICU admission and a total hospitalisation of > 10 days, similar to the criteria outlined in a previous investigation [24].

### Data collection and biomarker measurements

Existing comorbidities, demographics and concomitant medications were noted on arrival, and results from subsequent routine laboratory and microbiology tests recorded. CRP and lactate measurements were conducted at each respective site. Surplus blood samples were retrospectively batch tested for PCT and MR-proADM using a commercially available double sandwich immunoassay (KRYPTOR™, Thermo Fisher Scientific, Germany), with results made unavailable throughout patient enrolment and hospitalisation. Clinical scores including SIRS, SOFA, qSOFA, NEWS and CRB-65 were retrospectively calculated whenever possible. For the purposes of this analysis, the SOFA score was used as the reference standard due to its role in clinically characterising infected patients within the Third International Consensus Definitions for Sepsis and Septic Shock [2], whilst MR-proADM was taken as the index test. Sepsis was classified according to both previous and current definitions, with no differentiation made between sepsis, severe sepsis or septic shock subgroups (Sepsis-2) [4], or sepsis and septic shock subgroups (Sepsis-3) [2].

### Statistical analysis

Data were reported using mean (standard deviation) for the symmetrically distributed variable of age, and median [first quartile–third quartile] for the duration of total hospitalisation, biomarker and clinical score variables, which showed a skewed distribution. Differences in demographic and clinical characteristics with regard to 28-day mortality were assessed using the chi-square (*χ*^2^) test for categorical variables, Student’s *t* test for age, and the Mann-Whitney *U* test for all other continuous variables. Statistical procedures conducted for each analytical aim were as follows: *28-day mortality prediction*: Receiver operating characteristic (ROC) curves and areas under the curve (AUC) determined the parameter with the greatest predictive value, with 95% confidence intervals [95% CI] compared to determine significance. Youden’s criterion was used to establish optimal cut-off values, with sensitivity, specificity, negative and positive predictive values (NPV, PPV), negative and positive likelihood ratios (LR-, LR+) and diagnostic odds ratios (DOR) also reported. Kaplan-Meier curves identified patient subgroups using optimised or pre-determined cut-offs, with hazard ratios (HR) calculated between subgroups. Univariate and multivariate Cox regression models were performed to assess the association with survival time. Potential confounding variables were selected based on a univariate analysis (*p* value < 0.005 after applying a Bonferroni correction), and subsequently included in the multivariate analysis as adjusting variables. Survival time was censored at 28 days following ED presentation. Results were presented as the hazard ratio (HR) per 1 interquartile-range increase, with corresponding 95% CI. *Enrichment for uncomplicated infections and patients showing disease progression:* Patients were initially categorised into two groups based on cut-offs for each biomarker and score with respect to 28-day mortality. The parameter with the highest 28-day mortality predictive value was subsequently selected and patient populations further categorised to identify subgroups enriched for uncomplicated infections or patients showing disease progression. 28-day mortality and ICU admission rates, overall hospitalisation duration and the composite endpoints for uncomplicated infection and disease progression were compared between subgroups using the long-rank test for mortality, the chi-square (*χ*^2^) test for the composite scores and ICU admission, and the Mann-Whitney *U* test for the overall length of hospitalisation. *Hospitalisation and out-patient treatment decisions*: ROC and AUC were calculated for each parameter. Univariate and multivariate logistic regression assessed the association with hospitalisation decisions, with results presented as the odds ratio (OR) per 1 interquartile-range increase. Derivation and validation cohorts were subsequently pooled to derive improved 28-day mortality and hospitalisation cut-off values. A diagnostic meta-analysis was performed to calculate either the pooled hazard ratio for 28-day mortality or odds ratio for hospitalisation decisions for the biomarker or score with the highest derivation and validation cohort values. The presence of statistical heterogeneity between cohorts was assessed by the *I*^2^ test [25], with values of 25%, 50% or 75% regarded as indicative of low, moderate or high statistical heterogeneity, respectively. Post-test probabilities based on various pre-test probabilities (5% or 20% risk for 28-day mortality and hospitalisation) were illustrated using a Fagan nomogram [26]. A Bonferroni correction addressed the issue of multiple testing where appropriate. Optimised cut-offs for the biomarker or score with the highest predictive 28-day mortality and hospitalisation values were used to allocate patients to either virtual hospitalisation or out-patient treatment groups. Out-patients who later re-presented to the ED and were hospitalised were counted as readmissions. Both virtual and observed hospitalisation, out-patient treatment, readmission and 28-day mortality rates were subsequently calculated and compared. A *p* value < 0.05 was considered statistically significant. All data were analysed using the statistics software R (version 3.1.2), unless otherwise stated. Due to the exploratory nature of the derivation cohort, no *a priori* sample size calculation was performed.

## Results

### Patient characteristics

**Fig. 1:**
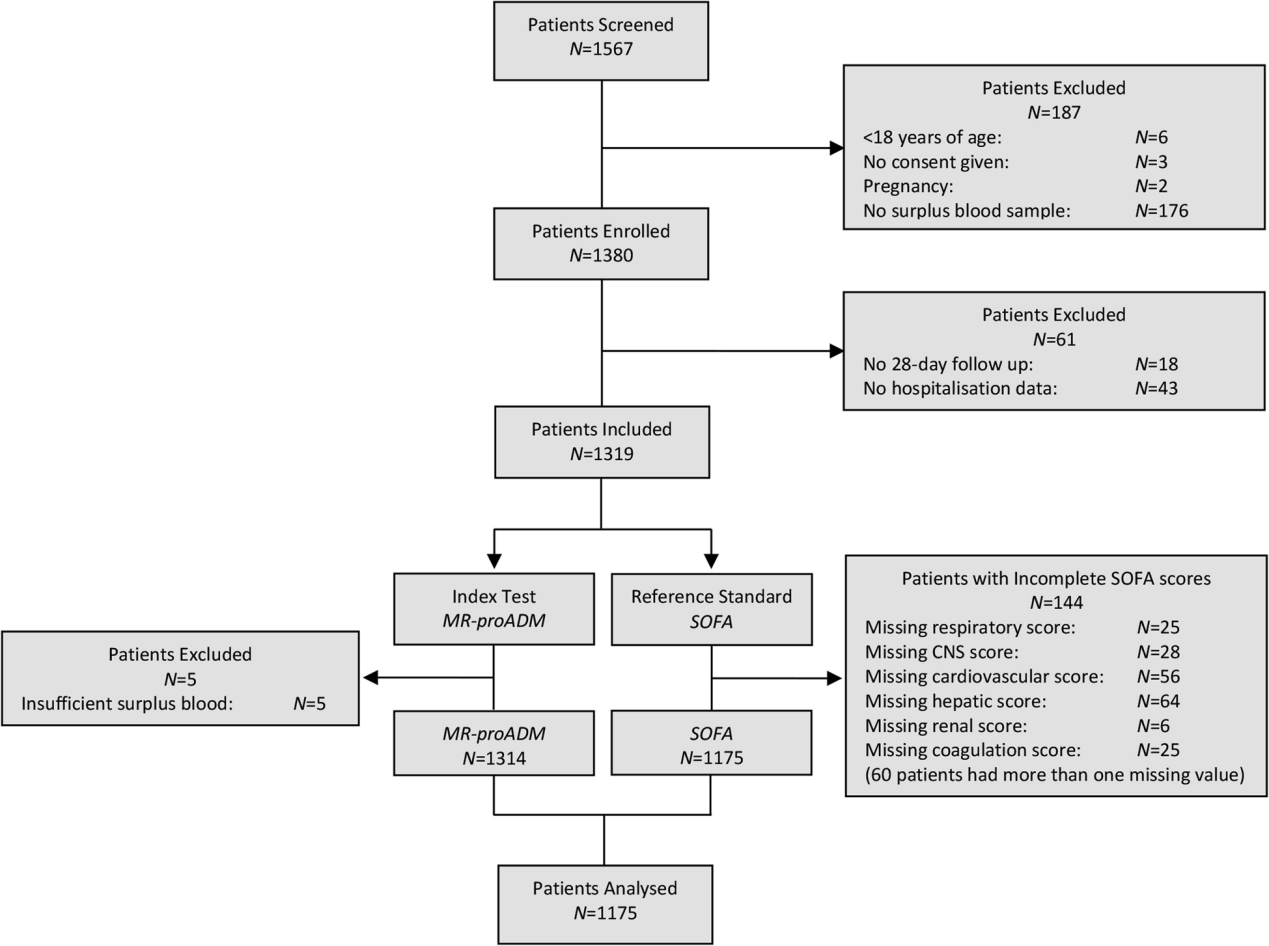
Flow chart describing the enrolment of patients. *CNS* central nervous system, *MR-proADM* mid-regional proadrenomedullin, *N* number, *SOFA* Sequential Organ Failure Assessment

A total of 1567 derivation patients were screened at baseline, with the exclusion of 392 patients predominantly due to missing information or insufficient surplus blood (Fig. 1). Thus, 1175 derivation patients were included in the final analysis, compared to 896 validation patients. The derivation cohort comprised of significantly older patients with a longer length of hospitalisation and a higher prevalence of suspected respiratory infection (all *p* < 0.001; Table 1), and is further described in Additional file 1: Table S1. Comparison of biomarker concentrations between cohorts found no differences between MR-proADM or PCT concentrations, whilst CRP values were significantly higher in the validation cohort (*p* < 0.001).

**Table 1:** Patient characteristics between derivation and validation cohorts

Patient characteristics	Derivation cohort (*N* = 1175)	Validation cohort (*N* = 896)	*p* value
Demographics			
Age (years) (mean, SD)	63.3 (20.9)	58.8 (21.0)	< 0.001
Male Sex (*N*, %)	592 (50.4%)	473 (52.8%)	0.266
Disposition			
Hospital admission (*N*, %)	915 (77.9%)	567 (76.2%)	0.397
Hospital length of stay (days) (median, Q1–Q3)	4 [1–9]	2 [0–6]	< 0.001
ICU admission (*N*, %)	32 (2.7%)	49 (5.5%)	0.001
28-day mortality (*N*, %)	84 (7.1%)	45 (5.0%)	0.098
Hospital mortality (*N*, %)	108 (9.2%)	38 (4.2%)	< 0.001
Comorbidities			
Cardiovascular disease (*N*, %)	363 (30.9%)	354 (39.5%)	0.003
Diabetes (*N*, %)	216 (18.4%)	142 (15.8%)	0.131
Malignancy (*N*, %)	228 (19.4%)	186 (20.8%)	0.445
Neurological disorders (*N*, %)	135 (11.5%)	67 (7.5%)	0.002
Respiratory disease (*N*, %)	378 (32.2%)	52 (5.8%)	< 0.001
Renal disease (*N*, %)	82 (7.0%)	169 (18.9%)	< 0.001
Suspected source of infection			
Fever of unknown origin (*N*, %)	98 (8.3%)	139 (18.4%)	< 0.001
Intra-abdominal (*N*, %)	158 (13.4%)	79 (10.5%)	0.051
Respiratory (*N*, %)	498 (42.4%)	258 (34.2%)	< 0.001
Skin and soft tissue (*N*, %)	96 (8.2%)	61 (8.1%)	0.943
Urogenital (*N*, %)	278 (23.7%)	164 (21.7%)	0.323
Other (*N*, %)	47 (4.0%)	53 (7.0%)	0.010
Biomarkers			
MR-proADM (nmol/L) (median, Q1–Q3)	1.09 [0.69–1.71]	1.03 [0.68–1.78]	0.888
PCT (ng/mL) (median, Q1–Q3)	0.17 [0.07–0.77]	0.14 [0.08–0.48]	0.244
CRP (mg/L) (median, Q1–Q3)	32 [10–120]	56 [15–142]	< 0.001

Values expressed in percentages (%) indicate the proportion of patients within each cohort for each variable. Data are presented as mean (standard deviation, SD) or median [first quartile (Q1)–third quartile (Q3)] where specified. The chi-square (*χ*^2^) test was used to determine significance between the cohorts for categorical variables, Student’s *t* test for the variable of age and Mann-Whitney *U* test for hospitalisation duration and biomarker concentrations. *CRP* C-reactive protein, *ICU* intensive care unit, *MR-proADM* mid-regional proadrenomedullin, *N* number, *PCT* procalcitonin

### 28-day mortality prediction

There were no significant differences in the all-cause 28-day mortality rate between the derivation (*N* = 84; 7.1%) and validation (*N* = 45; 5.0%) cohorts. Patient demographics and clinical characteristics according to survival are reported in Table 2 (derivation cohort) and Additional file 1: Table S2 (validation cohort), with patients further classified according to Sepsis-2 and Sepsis-3 definitions (Additional file 1: Table S3). All biomarkers and scores were significantly increased in the non-surviving patients of both cohorts (*p* < 0.001).

**Table 2:** Derivation cohort characteristics with regards to 28-day mortality

Patient characteristics	Total patient cohort (*N* = 1175)	Survivors (*N* = 1091)	Non-survivors (*N* = 84)	*p* value
Demographics				
Age (years) (mean, SD)	63.3 (20.9)	62.0 (20.9)	79.7 (11.6)	< 0.001
Male gender (*N*, %)	592 (50.4%)	543 (49.8%)	49 (58.3%)	0.130
Disposition				
Hospital admission (*N*, %)	915 (77.9%)	831 (76.2%)	84 (100.0%)	< 0.001
Hospital length of stay (days) (median, Q1–Q3)	4 [1–9]	4 [1–9]	11 [5–17]	< 0.001
ICU admission (*N*, %)	32 (2.7%)	18 (1.6%)	14 (16.7%)	< 0.001
Comorbidities				
Cardiovascular disease (*N*, %)	363 (30.9%)	316 (29.0%)	47 (56.0%)	< 0.001
Diabetes (*N*, %)	216 (18.4%)	196 (18.0%)	20 (23.8%)	0.183
Immunodeficiency (*N*, %)	64 (5.4%)	56 (5.1%)	8 (9.5%)	0.088
Liver disease (*N*, %)	31 (2.6%)	28 (2.6%)	3 (3.6%)	0.580
Malignancy (*N*, %)	228 (19.4%)	198 (18.1%)	30 (35.7%)	< 0.001
Neurological disorders (*N*, %)	135 (11.5%)	116 (10.6%)	19 (22.6%)	< 0.001
Respiratory disease (*N*, %)	378 (32.2%)	344 (31.5%)	34 (40.5%)	0.091
Renal disease (*N*, %)	82 (7.0%)	68 (6.2%)	14 (16.7%)	< 0.001
Infectious source				
Bone and Joint (*N*, %)	13 (1.1%)	13 (1.2%)	0 (0.0%)	0.315
Cardiac (*N*, %)	6 (0.5%)	5 (0.5%)	1 (1.2%)	0.364
Central nervous system (*N*, %)	13 (1.1%)	10 (0.9%)	3 (3.6%)	0.025
Fever of unknown origin (*N*, %)	98 (8.3%)	87 (8.0%)	11 (13.1%)	0.100
Foreign object (*N*, %)	5 (0.4%)	4 (0.4%)	1 (1.2%)	0.264
Intra-abdominal (*N*, %)	158 (13.4%)	153 (14.0%)	5 (6.0%)	0.007
Respiratory—lower (*N*, %)	413 (35.1%)	369 (33.8%)	44 (52.4%)	< 0.001
Respiratory—upper (*N*, %)	85 (7.2%)	85 (7.8%)	0 (0.0%)	0.008
Skin and soft tissue (*N*, %)	96 (8.2%)	89 (8.2%)	7 (8.3%)	0.901
Surgical-related (*N*, %)	10 (0.9%)	10 (0.9%)	0 (0.0%)	0.379
Urogenital (*N*, %)	278 (23.7%)	266 (24.4%)	12 (14.3%)	0.041
Microbiological findings				
Blood cultures taken (*N*, %)	888 (75.6%)	823 (75.4%)	65 (77.4%)	0.689
Positive blood cultures (*N*, %)	227 (19.3%)	205 (18.8%)	22 (26.2%)	0.099
Gram-positive bacteria (*N*, %)	120 (10.2%)	108 (9.9%)	12 (14.3%)	0.201
Gram-negative bacteria (*N*, %)	179 (15.2%)	166 (15.2%)	13 (15.5%)	0.949
Fungal cultures (*N*, %)	9 (0.8%)	8 (0.7%)	1 (1.2%)	0.643
Viral PCR (*N*, %)	40 (3.4%)	39 (3.6%)	1 (1.2%)	0.246
Other (*N*, %)	9 (0.8%)	8 (0.7%)	1 (1.2%)	0.830
Biomarkers and clinical scores				
MR-proADM (nmol/L) (median, Q1–Q3)	1.09 [0.69–1.71]	1.02 [0.67–1.59]	2.65 [1.81–4.67]	< 0.001
PCT (ng/mL) (median, Q1–Q3)	0.17 [0.07–0.77]	0.16 [0.07–0.61]	0.94 [0.23–3.12]	< 0.001
Lactate (mmol/L) (median, Q1–Q3)	1.60 [1.14–2.30]	1.55 [1.10–2.23]	2.40 [1.50–3.50]	< 0.001
CRP (mg/L) (median, Q1–Q3)	32 [10–120]	30 [10–112]	102 [28–178]	< 0.001
SIRS (points) (median, Q1–Q3)	2 [1–3]	2 [1–3]	3 [2–3]	< 0.001
SOFA (points) (median, Q1–Q3)	2 [0–3]	1 [0–3]	4 [2–6]	< 0.001
qSOFA (points) (median, Q1–Q3)	0 [0–1]	0 [0–1]	1 [1–2]	< 0.001
NEWS (points) (median, Q1–Q3)	4 [2–7]	4 [2–7]	8 [5–10]	< 0.001
CRB-65 (points) (median, Q1–Q3)	1 [0–2]	1 [0–1]	2 [1–2]	< 0.001

Values expressed in percentages (%) indicate either the proportion of the total patient cohort, surviving or non-surviving patients at 28 days for each variable, where applicable. Data are presented as mean (standard deviation, SD) or median [first quartile (Q1)–third quartile (Q3)] where appropriate. The chi-square (*χ*^2^) test was used to determine significance between surviving and non-surviving patients for categorical variables, Student’s *t* test for the variable of age, and Mann-Whitney *U* test for hospitalisation duration, biomarker and clinical score variables. *CRB-65* Severity score for community-acquired pneumonia, *CRP* C-reactive protein, *ICU* intensive care unit, *MR-proADM* mid-regional proadrenomedullin, *N* number, *NEWS* National Early Warning Score, *PCR* polymerase chain reaction, *PCT* procalcitonin, *qSOFA* quick Sequential Organ Failure Assessment, *SIRS* systemic inflammatory response syndrome, *SOFA* Sequential Organ Failure Assessment

**Fig. 2:**
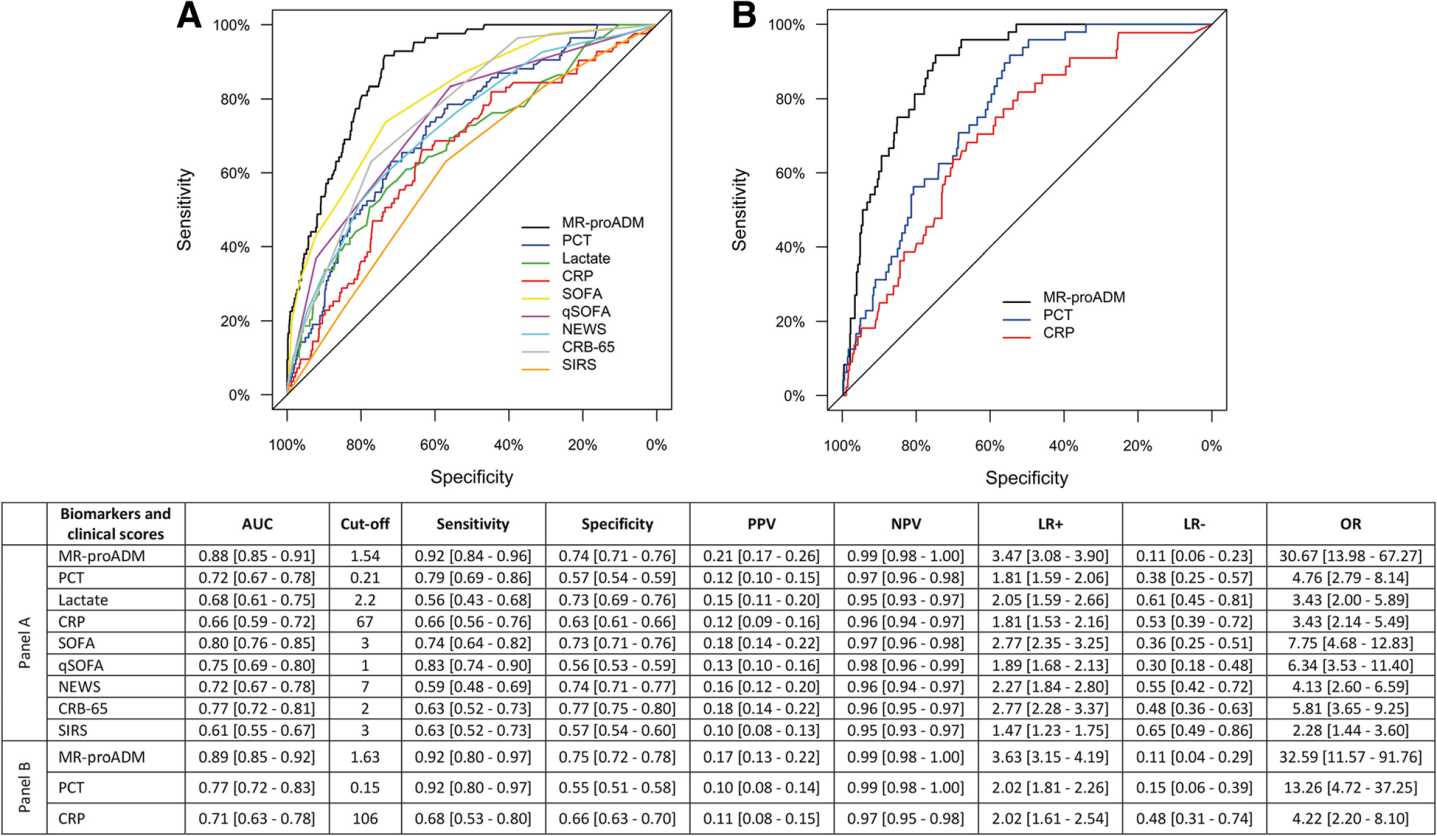
ROC curve and AUC analysis for 28-day mortality prediction within the derivation (**a**) and validation (**b**) cohorts following presentation to the emergency department. *AUC* area under the curve, *CRB-65* severity score for community-acquired pneumonia, *CRP* C-reactive protein, *LR-* negative likelihood ratio, *LR+* positive likelihood ratio, *MR-proADM* mid-regional proadrenomedullin, *NEWS* National Early Warning Score, *OR* diagnostic odds ratio, *NPV* negative predictive value, *PCT* procalcitonin, *PPV* positive predictive value, *qSOFA* quick Sequential Organ Failure Assessment, *ROC* receiver operating characteristic, *SIRS* systemic inflammatory response syndrome, *SOFA* Sequential Organ Failure Assessment

Univariate Cox regression analysis found that MR-proADM had the strongest association in predicting 28-day mortality in the derivation and validation cohorts (Table 3). In a multivariate analysis, the derivation cohort model was adjusted for the influence of age and existing cardiovascular, neurological, renal and malignancy comorbidities, with similar results found when the model was applied to the validation cohort (Table 4). AUC analysis across both cohorts found that MR-proADM had a significantly greater accuracy compared to other biomarkers and scores (Fig. 2). Application of the optimised derivation cut-off in the validation cohort is reported in Additional file 1: Table S4. Results were similar to those obtained in the derivation and validation cohorts using their respective optimised cut-offs. Pooling of the combined 2071 derivation and validation patients resulted in an identical cut-off to that of the derivation cohort (Additional file 1: Table S5), with meta-analysis reporting similar overall hazard ratios and a moderate degree of heterogeneity between cohorts (Additional file 1: Figure S1). Varying the pre-test prevalence of 28-day mortality (low mortality risk: 5%, or high mortality risk: 20%) resulted in high positive and low negative post-test probabilities for MR-proADM in each case (Additional file 1: Figure S2).

**Table 3:** Univariate Cox regression for the prediction of 28-day mortality in the derivation and validation cohorts

Biomarker or clinical score	Patients (*N*)	Mortality (*N*)	LR *χ*^2^	DF	*p* value	C-index	HR IQR [95% CI]
Derivation cohort							
MR-proADM	1175	84	166.4	1	< 0.001	0.869	5.4 [4.2–6.9]
PCT	1166	84	42.4	1	< 0.001	0.713	2.1 [1.7–2.6]
Lactate	746	59	25.3	1	< 0.001	0.678	2.2 [1.6–2.9]
CRP	1170	83	19.7	1	< 0.001	0.649	2.5 [1.6–3.8]
SIRS	965	84	12.2	1	< 0.001	0.640	1.9 [1.3–2.8]
SOFA	1175	84	83.5	1	< 0.001	0.827	2.6 [2.2–3.1]
qSOFA	1175	84	73.4	1	< 0.001	0.836	3.2 [2.5–4.0]
NEWS	1058	81	53.0	1	< 0.001	0.734	3.1 [2.3–4.2]
CRB-65	1175	84	75.8	1	< 0.001	0.838	2.6 [2.1–3.2]
Validation cohort							
MR-proADM	896	45	84.2	1	< 0.001	0.881	3.8 [2.9–5.0]
PCT	884	45	32.4	1	< 0.001	0.770	2.0 [1.6–2.5]
CRP	780	42	19.4	1	< 0.001	0.703	3.1 [1.7–5.6]

*CI* confidence interval, *CRB-65* severity score for community-acquired pneumonia, *CRP* C-reactive protein, *DF* degrees of freedom, *HR* hazard ratio, *IQR* interquartile range, *LR* likelihood ratio, *MR-proADM* mid-regional proadrenomedullin, *N* number, *NEWS* National Early Warning Score, *PCT* procalcitonin, *qSOFA* quick Sequential Organ Failure Assessment, *SIRS* systemic inflammatory response syndrome, *SOFA* Sequential Organ Failure Assessment

**Table 4:** Multivariate Cox regression for the prediction of 28-day mortality in the derivation and validation cohorts

Biomarker or clinical score	Patients (*N*)	Mortality (*N*)	LR *χ*^2^	DF	*p* value	C-index	HR IQR [95% CI]
Derivation cohort							
MR-proADM	1175	84	196.6	6	< 0.001	0.883	5.2 [3.9–6.9]
PCT	1166	84	112.0	6	< 0.001	0.813	2.0 [1.6–2.5]
Lactate	746	59	59.2	6	< 0.001	0.771	2.2 [1.6–3.0]
CRP	1170	83	97.3	6	< 0.001	0.787	2.6 [1.7–4.0]
SIRS	965	84	91.6	6	< 0.001	0.779	2.1 [1.4–3.0]
SOFA	1175	84	143.3	6	< 0.001	0.840	2.9 [2.4–3.7]
qSOFA	1175	84	117.7	6	< 0.001	0.825	2.5 [1.9–3.2]
NEWS	1058	81	105.2	6	< 0.001	0.803	2.5 [1.8–3.4]
CRB-65	1175	84	99.3	6	< 0.001	0.793	2.0 [1.5–2.5]
Validation cohort							
MR-proADM	896	45	114.6	6	< 0.001	0.899	3.7 [2.6–5.2]
PCT	884	45	80.7	6	< 0.001	0.847	1.6 [1.3–2.1]
CRP	780	42	75.2	6	< 0.001	0.837	2.4 [1.2–4.6]

Age, cardiovascular, neurological, renal and malignancy comorbidities were used as adjusting variables within the multivariate derivation cohort model, and subsequently applied to the validation cohort. *CI* confidence interval, *CRB-65* severity score for community-acquired pneumonia, *CRP* C-reactive protein, *DF* degrees of freedom, *HR* hazard ratio, *IQR* interquartile range, *LR* likelihood ratio, *MR-proADM* mid-regional proadrenomedullin, *N* number, *NEWS* National Early Warning Score, *PCT* procalcitonin, *qSOFA* quick Sequential Organ Failure Assessment, *SIRS* systemic inflammatory response syndrome, *SOFA* Sequential Organ Failure Assessment

**Fig. 3:**
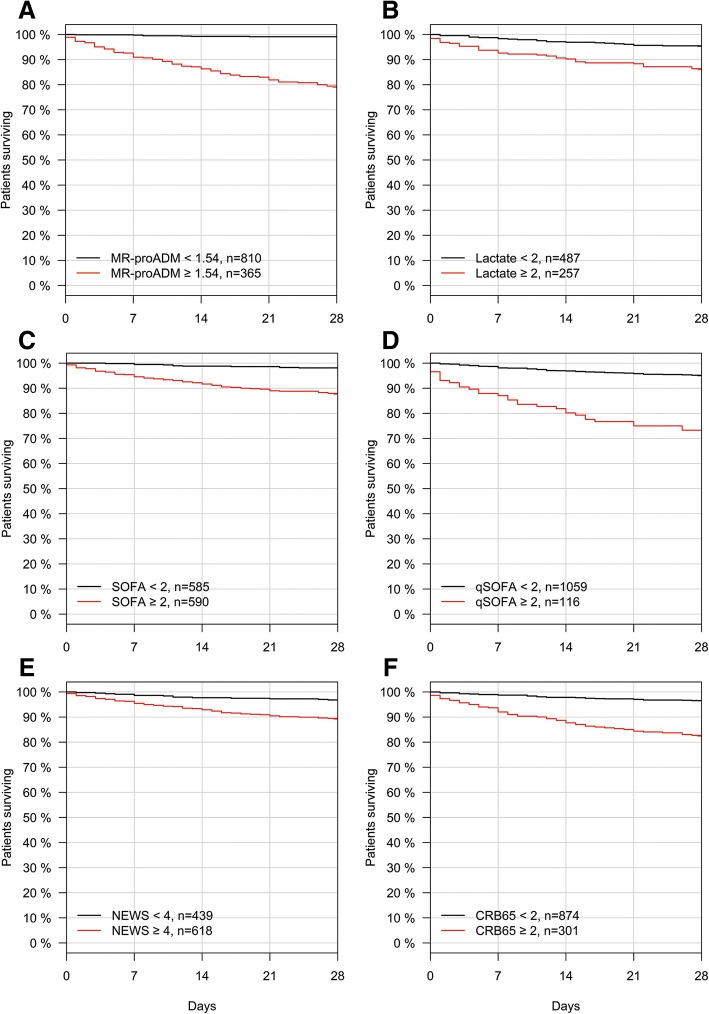
Kaplan-Meier analysis to identify disease severity subgroups using biomarkers and clinical scores within the derivation patient population according to MR-proADM (**a**), lactate (**b**), SOFA (**c**), qSOFA (**d**), NEWS (**e**) and CRB-65 (**f**) cut-offs. *CRB-65* severity score for community-acquired pneumonia, *MR-proADM* mid-regional proadrenomedullin, *NEWS* National Early Warning Score, *qSOFA* quick Sequential Organ Failure Assessment, *SOFA* Sequential Organ Failure Assessment

Kaplan-Meier curves using the optimised MR-proADM derivation cut-off could identify similar low and high disease severity subgroups within the derivation (low vs. high severity: *N* = 810 vs. 365; 0.9% vs. 21.1% mortality; *p* < 0.001; Fig. 3a) and validation (low vs. high severity: *N* = 612 vs. 284; 0.7% vs. 14.4% mortality; *p* < 0.001) cohorts, with comparable Cox regression analysis results (Additional file 1: Table S6). Identification of disease severity using other biomarkers and scores resulted in a lower discrimination and hazard ratio between subgroups (Fig. 3b–f; Additional file 1: Table S6).

### Enrichment for uncomplicated infections

**Fig. 4:**
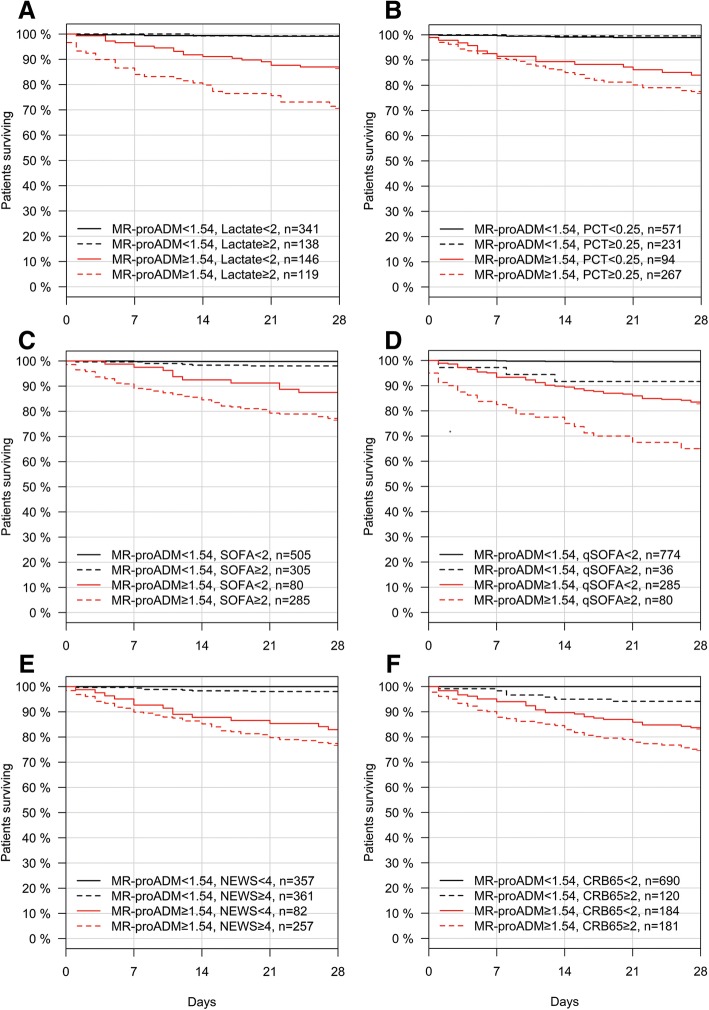
Kaplan-Meier analysis to identify patient populations enriched for either uncomplicated infections or further disease progression within the derivation cohort. Patients were stratified according to a combination of MR-proADM and lactate (**a**), PCT (**b**), SOFA (**c**), qSOFA (**d**), NEWS (**e**) and CRB-65 (**f**) cut-offs. *CRB-65* severity score for community-acquired pneumonia, *MR-proADM* mid-regional proadrenomedullin, *NEWS* National Early Warning Score, *PCT* procalcitonin, *qSOFA* quick Sequential Organ Failure Assessment, *SOFA* Sequential Organ Failure Assessment

Based on its high predictive value for 28-day mortality, MR-proADM was subsequently selected to further stratify patients following initial classification with other biomarkers and scores. The presence of low biomarker (PCT < 0.25 ng/mL, lactate < 2.0 mmol/L or CRP < 67 mg/L) or clinical score (SOFA < 2 points, qSOFA < 2 points, NEWS < 4 points or CRB-65 < 2 points) values resulted in a high number of uncomplicated infections (Additional file 1: Table S7), which could be further enriched using MR-proADM concentrations < 1.54 nmol/L, resulting in the identification of large patient populations with low mortality rates, low ICU admission rates, low lengths of hospitalisation and a higher number of uncomplicated infections according to the composite endpoint (Fig. 4; Additional file 1: Figure S3–8 and Table S8).

### Enrichment for patients at risk of disease progression

Conversely, the presence of low biomarker or score values with MR-proADM concentrations ≥ 1.54 nmol/L resulted in smaller patient populations, but with a significantly higher mortality and ICU admission rate, significantly longer length of hospitalisation, and a significantly higher number of disease progression events than found within the subgroups for low biomarker or score values and MR-proADM concentrations < 1.54 nmol/L (Fig. 4; Additional file 1: Table S9–10).

Even in patients where both SOFA and qSOFA values were < 2 points, MR-proADM concentrations were significantly higher in the non-surviving (*N* = 11; 2.02 [1.64–3.68] nmol/L) as opposed to surviving (*N* = 564; 0.76 [0.57–1.12] nmol/L; *p* < 0.001) patient population. The average time of death was 11 [9–16.5] days with no significant differences found in other standard laboratory parameters.

### Hospitalisation and out-patient treatment decisions

No significant differences in hospitalisation or out-patient treatment rates were found between the derivation (*N* = 915; 77.9% and *N* = 260; 22.1%) and validation (*N* = 567; 76.2% and *N* = 177; 23.8%) cohorts following ED presentation, with patients selected for out-patient treatment having similar 14-day rehospitalisation (derivation: *N* = 10; 5.3% vs. validation: *N* = 9; 5.1%) and 28-day mortality (derivation: *N* = 0; 0.0% vs. validation: *N* = 1; 0.6%) rates.

**Fig. 5:**
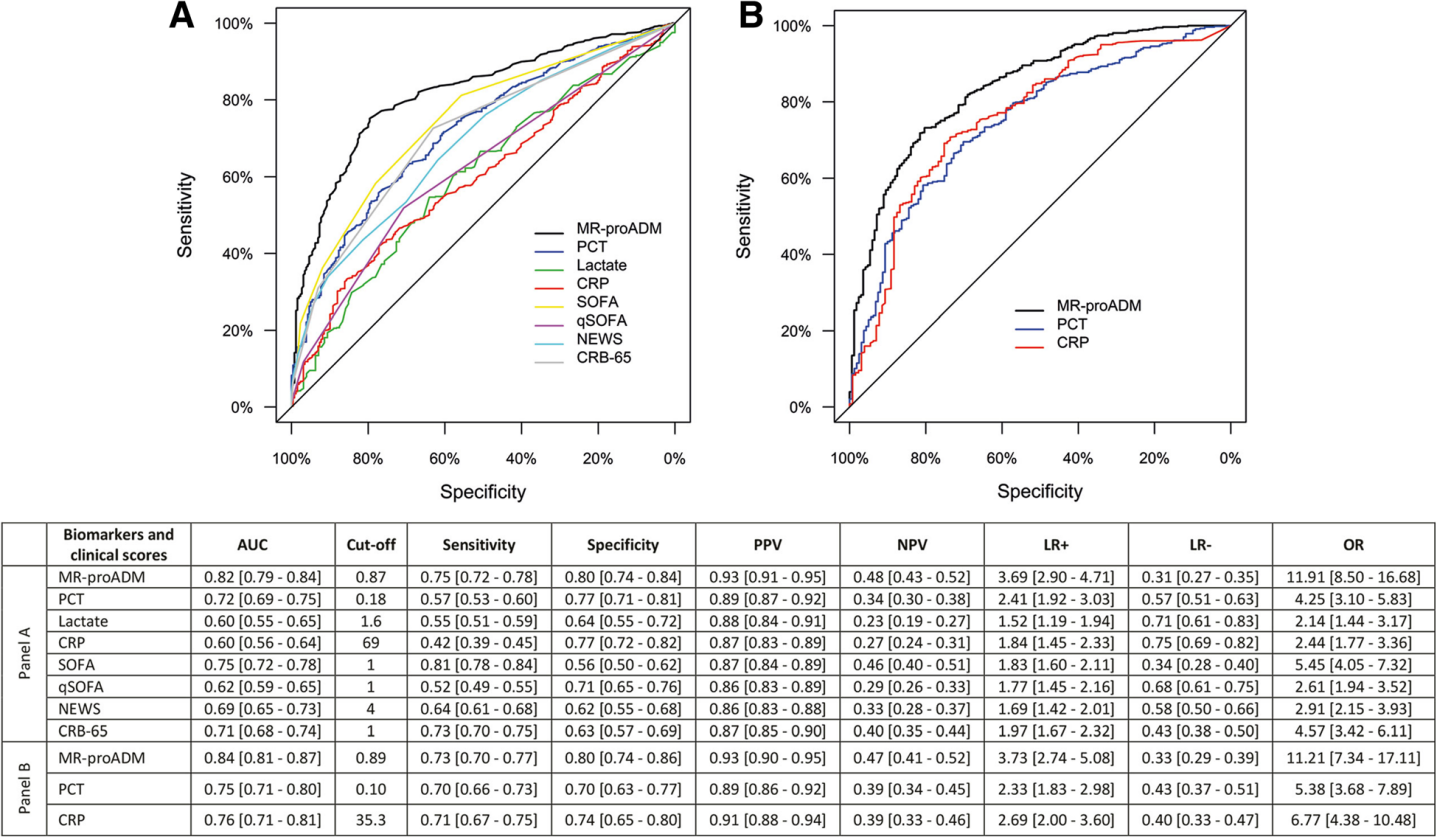
ROC curve and AUC analysis for hospitalisation decisions within the derivation (**a**) and validation (**b**) cohorts following presentation to the Emergency Department. *AUC* area under the curve, *CRB-65* severity score for community-acquired pneumonia, *CRP* C-reactive protein, *LR-* negative likelihood ratio, *LR+* positive likelihood ratio, *MR-proADM* mid-regional proadrenomedullin, *NEWS* National Early Warning Score, *NPV* negative predictive value, *OR* diagnostic odds ratio, *PCT* procalcitonin, *PPV* positive predictive value, *ROC* receiver operating characteristic, *qSOFA* quick Sequential Organ Failure Assessment, *SOFA* Sequential Organ Failure Assessment

Univariate logistic regression found that MR-proADM had the strongest association with hospitalisation decisions across both cohorts (Additional file 1: Table S11). In a multivariate analysis, the derivation cohort was adjusted for the same confounding variables as within the 28-day mortality model, yielding similar results for both cohorts (Additional file 1: Table S12). Comparable accuracies were obtained for derivation and validation AUC analyses (Fig. 5), with results using the optimised derivation cut-off in the validation cohort reported in Additional file 1: Tables S13–14. Pooling of the combined 2071 derivation and validation patients resulted in an identical cut-off to that of the validation cohort (Additional file 1: Table S15), with meta-analysis reporting similar overall odds ratios and a high degree of heterogeneity between cohorts (Additional file 1: Figure S9). Varying the pre-test prevalence for patient hospitalisation (low hospitalisation risk: 5%, or high hospitalisation risk: 20%) resulted in both high positive and low negative post-test probabilities for MR-proADM in each case (Additional file 1: Figure S10).

A total of 436 (37.1%) derivation and 362 (40.4%) validation patients had MR-proADM values below the optimised hospitalisation derivation cut-off (< 0.87 nmol/L), representing a potential increase in the derivation and validation out-patient populations of 15.0% and 16.6%, respectively. In addition, both subgroups had lower 14-day readmission rates compared to the actual out-patient population and no mortalities up to 28 days (Additional file 1: Figure S11–12). Conversely, application of the optimised derivation MR-proADM cut-off would have resulted in the hospitalisation of 53 (20.4%) derivation and 44 (24.9%) validation patients initially deemed suitable for out-patient treatment, including 7 (70.0%) derivation and 4 (44.4%) validation out-patients who presented to the emergency department an average of 1 day later and were subsequently hospitalised.

## Discussion

In this derivation and validation analysis of 2071 suspected infection patients presenting to 9 emergency departments across Europe and the USA, MR-proADM measurement at presentation could accurately assess disease severity and identify specific patient populations based on the likelihood of subsequent disease progression. This is of particular importance in patients with few pathophysiological signs and symptoms, as indicated by low SOFA, qSOFA or NEWS scores, where initial treatment may either be withheld, delayed or insufficient. Our study therefore, for the first time, highlights the use of MR-proADM in potentially identifying this patient population in order to initiate appropriate treatment strategies at the earliest opportunity.

An early and accurate identification of this key patient demographic, however, may be complicated by the lack of pathognomonic symptoms and the highly complex, heterogeneous and multifaceted host response to infection [27]. An early diagnosis of developing sepsis therefore invariably requires a complex clinical investigation incorporating numerous factors such as presenting symptoms, physician judgement, and standard laboratory and biomarker tests. Accordingly, earlier indicators of deteriorating host response are essential in order to provide relevant information at the earliest opportunity possible [19]. In this respect, MR-proADM is an interesting biomarker candidate, with previous studies showing increased concentrations in response to deteriorating microcirculatory integrity and resulting capillary leak, thus reflecting the early stages of developing organ dysfunction [28–31]. An early assessment of microcirculatory function may therefore contribute significant information as part of an initial multi-modal clinical examination, and provide a more accurate method of assessing disease progression and the efficacy of therapeutic interventions compared to the use of conventional biomarkers or scoring systems [30].

Based on the results of this study, two clinically important uses for MR-proADM can be proposed: (i) an early escalation of treatment in patients with MR-proADM concentrations ≥ 1.5 nmol/L, thus identifying an already high level of disease severity or a high potential for further development and progression, and (ii) a decreased number of hospital admissions allowing a safe increase in out-patient treatment in patients with MR-proADM concentrations < 0.9 nmol/L.

First, an early identification of further disease development and progression in patients with uncomplicated infections and minimal organ dysfunction is crucial in order to initiate, escalate or intensify treatment at the earliest opportunity. Our results identified a large patient population with few clinical or laboratory signs which would prompt an immediate and urgent therapeutic response. The presence of elevated MR-proADM concentrations in a subset of these patients, however, resulted in long lengths of hospitalisation, a high likelihood of mortality, increased ICU admission rates, and a high number of patients satisfying the composite endpoint for disease progression, compared to those with low MR-proADM levels. Such findings may facilitate specific interventions such as the rapid administration of antibiotics and fluids, the use of adjunctive sepsis therapies, or additional diagnostic testing in order to prevent potential cases of under-treatment or inappropriate discharge. In addition, a more personalised and tailored therapeutic approach may be most beneficial in patients with the highest MR-proADM concentrations (e.g. > 2.75 nmol/L [19]), with the early admission onto a high dependency or intensive care unit to initiate aggressive therapeutic strategies, such as those targeting extravascular fluid accumulation, potentially decreasing further organ dysfunction or progression towards multiple organ failure [20, 32]. Interestingly, MR-proADM concentrations > 2.75 nmol/L in our study (*N* = 126; 10.7%) resulted in a 28-day mortality rate of 30.2%, similar to the 32.5% found in the intensive care study of Elke et al. [19] in patients with corresponding concentrations (*N* = 759; 73.7%).

Few studies have reported similar findings to ours. Indeed, numerous investigations have focussed on mortality and adverse event prediction in patients with community-acquired pneumonia (CAP), comparing MR-proADM performance to clinical scores such as CURB-65 and the Pneumonia Severity Index (PSI), with a moderate to good discriminatory performance found for both endpoints and similar cut-offs compared to our analysis [33–38]. Similar results were also reported for mortality prediction outside the intensive care setting using SOFA and qSOFA scores in the recent sepsis-3 definitions [2], thus partially confirming and validating results from our analysis.

Findings observed in our study may, in part, be explained by the rapidly induced kinetical profile of MR-proADM in response to LPS addition, compared to other parameters such as procalcitonin and C-reactive protein [16]. Initial microcirculatory dysfunction due to infection is likely to drive the expected physiological development towards organ dysfunction and ultimate multiple organ failure [39]. Hence, measurement of MR-proADM values upon ED presentation may provide an early indication concerning potential disease progression [30]. Similar findings have also been observed in an intensive care setting in patients with high MR-proADM concentrations and initially low or moderate levels of organ dysfunction that progressed towards sepsis-related multiple organ failure [19, 20, 40]. Indeed, continuously elevated concentrations, despite decreasing PCT levels over the first 24 h of treatment, indicated a high likelihood of subsequent treatment failure and disease progression, thus providing an early and independent prompt with which to change or modify treatment [19]. The use of MR-proADM to identify the likelihood of infection-related disease progression may therefore be of significant clinical value irrespective of clinical setting or initial disease severity.

Second, a more accurate identification of uncomplicated infections with a low risk of further progression may improve initial hospitalisation and out-patient treatment decision-making. Our results found a similar performance of MR-proADM within both the derivation and validation cohorts, with comparable increases in outpatient numbers, a lack of subsequent mortality and decreased rehospitalisation rates.

Surprisingly, only few studies with relatively small patient populations have previously investigated the accuracy of hospitalisation and out-patient treatment decisions in infected patients using MR-proADM. Of these, Travaglino et al. [41] observed a poor performance in 128 patients with high fever and mixed infections, as opposed to the high discriminatory performance found by Starre et al. [42] in 321 urinary tract infection (UTI) patients. A recent secondary analysis of 313 UTI patients by Stalenhoef et al. [24] found similar results to our analysis using a comparable cut-off, with increased out-patient treatment, no mortality and fewer cases of subsequent rehospitalisation. Conversely, Albrich et al. [43] tested a novel algorithm combining CURB-65 and MR-proADM values in an interventional setting of 313 lower respiratory tract infection (LRTI) patients [44], resulting in significantly increased out-patient numbers and decreased overruling and readmission rates compared to a control group triaged using CURB-65 alone [45]. Results from our study are therefore derived from the largest sample size of patients with a suspected infection presenting to the ED with initial hospitalisation and out-patient treatment decision data to date. The high performance of MR-proADM as a stand-alone parameter as opposed to in combination with a clinical score may facilitate easier use in high patient settings such as the ED, although further observational and interventional studies with similarly large patient populations are required to confirm and validate our findings. The generation of corresponding health economic data would also be beneficial in highlighting potential cost savings from increased out-patient treatment.

Our study has several limitations. Firstly, the absence of subsequent biomarker and clinical score measurements after hospital admission only allow assumptions to be made concerning disease progression and sepsis development, according to current definitions [2]. Similar studies investigating MR-proADM kinetics in LRTI patients over 72 h have previously shown a decreased survival probability in patients with increasing or continuously elevated concentrations [46]. Nevertheless, future studies should be designed considering additional variables such as SOFA score kinetics between admission and either 48 or 72 h to investigate developing organ dysfunction and sepsis progression as relevant endpoints. In addition, the inclusion of hospitalisation duration as a variable in the composite endpoint for identifying uncomplicated infections and disease progression may not take numerous time-dependent internal and external clinical factors into account, and could result in different findings if an alternative discriminatory value is used. Secondly, clinical scores in the validation cohort could not be calculated due to the absence of key clinical data, thus, a direct comparison between cohorts was not possible. Finally, mortality and hospitalisation prevalence in other hospitals and clinical settings may significantly differ with one another, leading to the calculation of different negative and positive predictive values, and resulting in study results which are not directly transferrable.

We note several strengths. Firstly, the comparative use of two large, independent, multicentre patient populations ensured a high degree of internal validity, with similar patient demographics between cohorts. Nevertheless, future studies would greatly benefit from the inclusion of further EDs from alternative geographies, different income-settings and hospitals with significantly different triage procedures, in order to rule-out any significant influence on results and increase the reproducibility of the findings. Secondly, the use of optimised derivation MR-proADM cut-offs resulted in similar findings across both cohorts with regard to the identification of disease severity and out-patient treatment, strengthening its potential use in both areas.

## Conclusions

MR-proADM measurement upon ED presentation may allow for an early identification of patients with suspected infection who may suffer from subsequent disease progression. Conversely, a more accurate identification of those with uncomplicated infections and the rule-out of further disease progression may facilitate an increased rate of out-patient treatment with a low number of subsequent readmissions. Incorporation of MR-proADM into an early sepsis management protocol may therefore aid rapid clinical decision making and subsequent treatment decisions in the emergency department, thus improving personalised sepsis strategies.

## Additional files

Additional file 1: **Table S1.** Initial clinical diagnoses and infectious source (derivation cohort). **Table S2.** 28-day mortality validation cohort characteristics. **Table S3.** Sepsis-2 and sepsis-3 classification (derivation cohort). **Table S4.** Application of the 28-day mortality derivation MR-proADM cut-off (validation cohort). **Table S5.** Pooled derivation and validation cohorts for 28-day mortality prediction. **Table S6.** Cox regression between high and low severity populations (derivation and validation cohorts). **Table S7.** Uncomplicated infection and disease progression events in the low 28-day mortality biomarker/score value subgroups. **Table S8.** Subgroups with low MR-proADM and low biomarker/score values showing enrichment for uncomplicated infections. **Table S9.** Subgroups with high MR-proADM and low biomarker/score values showing enrichment for disease progression events. **Table S10.** Significance between uncomplicated infection or disease progression subgroups for hospitalisation duration, 28-day mortality and ICU admission rates. **Table S11.** Univariate logistic regression for hospitalisation decisions. **Table S12.** Multivariate logistic regression for hospitalisation decisions. **Table S13.** Application of the optimised derivation MR-proADM cut-off for hospitalisation (derivation and validation cohort). **Table S14.** Derivation and validation logistic regression for hospitalisation using optimised derivation cut-offs. **Table S15.** Combined derivation and validation cohort meta-analysis for hospitalisation decisions. **Figure S1.** Meta-analysis assessing derivation and validation heterogeneity for 28-day mortality. **Figure S2.** Fagan nomogram calculating post-test probabilities for 28-day mortality. **Figure S3.** Kaplan-Meier using the optimised derivation lactate cut-off. **Figure S4.** Kaplan-Meier using the optimised derivation PCT cut-off. **Figure S5.** Kaplan-Meier using the optimised derivation qSOFA cut-off. **Figure S6.** Kaplan-Meier using the optimised derivation SOFA cut-off. **Figure S7.** Kaplan-Meier using the optimised derivation SIRS cut-off. **Figure S8.** Kaplan-Meier using the optimised derivation NEWS cut-off. **Figure S9.** Meta-analysis assessing derivation and validation heterogeneity for hospitalisation decisions. **Figure S10.** Fagan nomogram calculating post-test probabilities for hospitalisation decisions. **Figure S11.** Conventional and virtual MR-proADM guided-triage (derivation cohort). **Figure S12.** Conventional and virtual MR-proADM guided-triage (validation cohort). (DOCX 1318 kb)

## Abbreviations

AUC: Area under the curve
CAP: Community-acquired pneumonia
CI: Confidence interval
CRB-65 and CURB-65: Severity scores for community-acquired pneumonia
CRP: C-reactive protein
ED: Emergency department
HR: Hazard ratio
IQR: Interquartile range
LPS: Lipopolysaccharide
LRTI: Lower respiratory tract infection
MR-proADM: Mid-regional proadrenomedullin
N: Number
NEWS: National Early Warning Score
OR: Odds ratio
PCT: Procalcitonin
PSI: Pneumonia Severity Index
qSOFA: Quick Sequential Organ Failure Assessment
ROC: Receiver operating characteristic
SIRS: Systemic Inflammatory Response Syndrome
SOFA: Sequential Organ Failure Assessment
UTI: Urinary tract infection
