# Correction to: The early identification of disease progression in patients with suspected infection presenting to the emergency department: a multi-centre derivation and validation study

**DOI:** 10.1186/s13054-019-2536-0

**Published:** 2019-07-15

**Authors:** Kordo Saeed, Darius Cameron Wilson, Frank Bloos, Philipp Schuetz, Yuri van der Does, Olle Melander, Pierre Hausfater, Jacopo M. Legramante, Yann-Erick Claessens, Deveendra Amin, Mari Rosenqvist, Graham White, Beat Mueller, Maarten Limper, Carlota Clemente Callejo, Antonella Brandi, Marc-Alexis Macchi, Nicholas Cortes, Alexander Kutz, Peter Patka, María Cecilia Yañez, Sergio Bernardini, Nathalie Beau, Matthew Dryden, Eric C. M. van Gorp, Marilena Minieri, Louisa Chan, Pleunie P. M. Rood, Juan Gonzalez del Castillo

**Affiliations:** 1 Department of Microbiology, Hampshire Hospitals NHS Foundation Trust, Winchester and Basingstoke, UK; 2 School of Medicine, University of Southampton, Southampton, UK; 3 B·R·A·H·M·S GmbH, Hennigsdorf, Germany; 4 Department of Anesthesiology and Intensive Care Medicine, Jena University Hospital, Jena, Germany; 5 Center for Sepsis Control & Care (CSCC), Jena University Hospital, Jena, Germany; 6 Division of General and Emergency Medicine, University Department of Medicine, Kantonsspital Aarau, Switzerland; 7 Medical Faculty of the University of Basel, Basel, Switzerland; 8 Department of Emergency Medicine, Erasmus University Medical Center, Rotterdam, Netherlands; 9 Department of Internal Medicine, Skåne University Hospital, Malmö, Sweden; 10 Department of Clinical Sciences Malmö, Lund University, Lund, Sweden; 11 Emergency Department Hôpital Pitié-Salpêtrière, Assistance Publique - Hôpitaux de Paris and Sorbonne Universités GRC-14 BIOSFAST and INSERM UMR-S 1166, Paris, France; 12 Emergency Department, Policlinico Tor Vergata, Rome, Italy; 13 Department of Medical Systems, Universita di Tor Vergata, Rome, Italy; 14 Department of Emergency Medicine, Monaco Princess Grace Hospital, Monaco, France; 15 Department of Critical Care, Morton Plant Hospital, 300 Pinellas Street, Clearwater, FL 33756 USA; 16 Infectious Disease Unit, Skåne University Hospital, Malmö, Sweden; 17 Department of Blood Sciences, Hampshire Hospitals NHS Foundation Trust, and Basingstoke, Winchester, UK; 18 Department of Rheumatology and Clinical Immunology, University Medical Center, Utrecht University, Utrecht, Netherlands; 19 Emergency Department, Hospital Clínico San Carlos, Madrid, Spain; 20 Gibraltar Health Authority, St Bernard’s Hospital, Gibraltar, UK; 21 Department of Laboratory Medicine, Policlinico Tor Vergata, Rome, Italy; 22 Department of Experimental Medicine, University of Rome Tor Vergata, Rome, Italy; 23 Rare and Imported Pathogen Laboratories, Public Health England, Porton Down, UK; 24 Department of Internal Medicine, Erasmus University Medical Center, Rotterdam, Netherlands; 25 Department of Viroscience, Erasmus University Medical Center, Rotterdam, Netherlands; 26 Department of Accident and Emergency, Hampshire Hospitals NHS Foundation Trust, and Basingstoke, Winchester, UK; 27 Emergency Department, Instituto de Investigación Sanitaria (IdISSC), Hospital Clínico San Carlos, Madrid, Spain

**Correction to: Crit Care**

**https://doi.org/10.1186/s13054-019-2329-5**

In the publication of this article [1], there are two errors in contributing author affiliations. This has now been included in this correction article.

The error in affiliation 20:

Gibraltar Health Authority, St Bernard’s Hospital, Gibraltar, Spain

Should instead read:

Gibraltar Health Authority, St Bernard’s Hospital, Gibraltar, UK

The error: the contributing author Marilena Minieri was only affiliated with 21 but is in fact affiliated with ^21^ and ^22^:

21. Department of Laboratory Medicine, Policlinico Tor Vergata, Rome, Italy.

22. Department of Experimental Medicine, University of Rome Tor Vergata, Rome, Italy.
