# Supplementary material for: The early identification of disease progression in patients with suspected infection presenting to the emergency department: a multi-centre derivation and validation study
Source: Crit Care. 2019 Feb 8;23:40. doi: 10.1186/s13054-019-2329-5 (PMC6368690; doi:10.1186/s13054-019-2329-5)
Supplement: Supplementary file 1 — Table S1. Initial clinical diagnoses and infectious source (derivation cohort). Table S2. 28-day mortality validation cohort characteristics. Table S3. Sepsis-2 and sepsis-3 classification (derivation cohort). Table S4. Application of the 28-day mortality derivation MR-proADM cut-off (validation cohort). Table S5. Pooled derivation and validation cohorts for 28-day mortality prediction. Table S6. Cox regression between high and low severity populations (derivation and validation cohorts). Table S7. Uncomplicated infection and disease progression events in the low 28-day mortality biomarker/score value subgroups. Table S8. Subgroups with low MR-proADM and low biomarker/score values showing enrichment for uncomplicated infections. Table S9. Subgroups with high MR-proADM and low biomarker/score values showing enrichment for disease progression events. Table S10. Significance between uncomplicated infection or disease progression subgroups for hospitalisation duration, 28-day mortality and ICU admission rates. Table S11. Univariate logistic regression for hospitalisation decisions. Table S12. Multivariate logistic regression for hospitalisation decisions. Table S13. Application of the optimised derivation MR-proADM cut-off for hospitalisation (derivation and validation cohort). Table S14. Derivation and validation logistic regression for hospitalisation using optimised derivation cut-offs. Table S15. Combined derivation and validation cohort meta-analysis for hospitalisation decisions. Figure S1. Meta-analysis assessing derivation and validation heterogeneity for 28-day mortality. Figure S2. Fagan nomogram calculating post-test probabilities for 28-day mortality. Figure S3. Kaplan-Meier using the optimised derivation lactate cut-off. Figure S4. Kaplan-Meier using the optimised derivation PCT cut-off. Figure S5. Kaplan-Meier using the optimised derivation qSOFA cut-off. Figure S6. Kaplan-Meier using the optimised derivation SOFA cut-off. Figure S7. Kaplan-Meier using the [file 13054_2019_2329_MOESM1_ESM.docx]

**Additional File 1**

**The early identification of disease progression in patients with suspected infection presenting to the emergency department: a multi-centre derivation and validation study**

***Short title: Early identification of infectious disease progression***

**Kordo Saeed MB ChB FRCPath^1,2^, Darius Cameron Wilson PhD^3^, Frank Bloos MD PhD^4,5^, Philipp Schuetz MD^6,7^, Yuri van der Does MD PhD^8^, Olle Melander MD PhD^9,10^, Pierre Hausfater MD^11^, Jacopo Legramante MD^12,13^, Yann-Erick Claessens MD PhD^14^, Deveendra Amin MD^15^, Mari Rosenqvist MD^16,17^, Graham White BSc MSc^18^, Beat Mueller MD^6,7^, Maarten Limper MD PhD^19^, Carlota Clemente Callejo MD^20^, Antonella Brandi MD^12^, Marc-Alexis Macchi MD^14^, Nicolas Cortes MB BD FRCPath^1,2^, Alexander Kutz MD^6^, Peter Patka MD PhD^8^, María Cecilia Yañez MD^20^, Sergio Bernardini MD^21^, Nathalie Beau MD^14^, Matthew Dryden MD FRCPath^1,2,22^, Eric C.M. van Gorp MD PhD^23,24^, Marilena Minieri PhD^21^, Louisa Chan MB BS^25^, Pleunie P.M. Rood MD PhD^8^, Juan Gonzalez del Castillo MD PhD^26^**

1. Department of Microbiology, Hampshire Hospitals NHS Foundation Trust, Winchester and Basingstoke, UK
2. University of Southampton, School of Medicine, Southampton, UK
3. B·R·A·H·M·S GmbH, Hennigsdorf, Germany
4. Dept. of Anesthesiology and Intensive Care Medicine, Jena University Hospital, Jena, Germany
5. Center for Sepsis Control & Care (CSCC), Jena University Hospital, Jena, Germany
6. Division of General and Emergency Medicine, University Department of Medicine, Kantonsspital Aarau, Switzerland
7. Medical Faculty of the University of Basel, Basel, Switzerland
8. Department of Emergency Medicine, Erasmus University Medical Center, Rotterdam, Netherlands
9. Department of Internal Medicine, Skåne University Hospital, Malmö, Sweden
10. Department of Clinical Sciences Malmö, Lund University, Sweden
11. Emergency Department, Groupe Hospitalier Pitié-Salpêtrière Assistance, Publique-Hôpitaux de Paris (APHP), Paris, France
12. Emergency Department, Policlinico Tor Vergata, Roma, Italy
13. Department of Medical Systems, Universita di Tor Vergata, Roma, Italy
14. Department of Emergency Medicine, Monaco Princess Grace Hospital, Monaco, France
15. Department of Critical Care, Morton Plant Hospital, 300 Pinellas Street, Clearwater, FL 33756, USA
16. Infectious Disease Unit, Skåne University Hospital, Malmö, Sweden
17. Department of Clinical Sciences Malmö, Lund University, Sweden
18. Department of Blood Sciences, Hampshire Hospitals NHS Foundation Trust, Winchester and Basingstoke, UK
19. Department of Rheumatology and Clinical Immunology, University Medical Center, Utrecht University, Utrecht, Netherlands
20. Emergency Department, Hospital Clínico San Carlos, Madrid, Spain
21. Laboratory of Clinical Biochemistry, Department of Laboratory Medicine, Policlinico Tor Vergata, Roma, Italy
22. Rare and Imported Pathogen Laboratories, Public Health England, Porton Down, UK
23. Department of Internal Medicine, Erasmus University Medical Center, Rotterdam, Netherlands
24. Department of Viroscience, Erasmus University Medical Center, Rotterdam, Netherlands
25. Department of accident and emergency, Hampshire Hospitals NHS Foundation Trust, Winchester and Basingstoke, UK
26. Emergency Department, Instituto de Investigación Sanitaria (IdISSC), Hospital Clínico San Carlos, Madrid, Spain

**Corresponding author:** Dr. Kordo Saeed, Royal Hampshire County Hospital, Romsey Road, Winchester, SO22 5DG, England; Tel: +44 196 2825927; E-mail: [Kordosaeed@nhs.net](mailto:Kordosaeed@nhs.net)

**Contents**

**Supplementary Tables.....................................................................................................................3**

**Table S1. Initial clinical diagnoses and suspected infectious source in the derivation cohort**

**Table S2**. **Validation cohort characteristics with regards to 28-day mortality**

**Table S3**. **Sepsis-2 and sepsis-3 classification in the derivation cohort**

**Table S4**. **Application of the 28-day mortality derivation MR-proADM cut-off in the validation cohort**

**Table S5**. **Pooled derivation and validation cohorts for 28-day mortality prediction using MR-proADM**

**Table S6**. **Cox regression analysis between high and low severity populations for biomarkers and scores in the derivation and validation cohorts, using optimised or pre-established cut-offs**

**Table S7**. **Uncomplicated infection and disease progression events in the patient subgroup with biomarker or score values below their respective 28-day mortality cut-offs**

**Table S8**. **Patient subgroups with MR-proADM concentrations <1.54 nmol/L, combined with low biomarker or score values, showing enrichment for uncomplicated infections**

**Table S9**. **Patient subgroups with MR-proADM concentrations ≥1.54 nmol/L, combined with low biomarker or score values, showing enrichment for disease progression events**

**Table S10**. **Tests for significance in hospitalisation duration, 28-day mortality and ICU admission rates between subgroups enriched for uncomplicated infections or disease progression**

**Table S11**. **Derivation and validation univariate logistic regression for hospitalisation decisions**

**Table S12**. **Derivation and validation multivariate logistic regression for hospitalisation decisions**

**Table S13**. **Application of the optimised derivation MR-proADM cut-off for hospitalisation in the derivation and validation cohort**

**Table S14**. **Derivation and validation logistic regression analysis for hospitalisation using the optimised derivation cut-offs**

**Table S15**. **Meta-analysis of the combined derivation and validation cohorts for hospitalisation decisions using MR-proADM**

**Supplementary Figures..................................................................................................................10**

**Figure S1**. **Meta-analysis to assess derivation and validation heterogeneity for 28-day mortality**

**Figure S2. Fagan nomogram for the calculation of post-test probabilities for 28-day mortality**

**Figure S3**. **Kaplan-Meier analysis using the optimised derivation lactate cut-off to identify severity**

**Figure S4**. **Kaplan-Meier analysis using the optimised derivation PCT cut-off to identify severity**

**Figure S5**. **Kaplan-Meier analysis using the optimised derivation qSOFA cut-off to identify severity**

**Figure S6**. **Kaplan-Meier analysis using the optimised derivation SOFA cut-off to identify severity**

**Figure S7**. **Kaplan-Meier analysis using the optimised derivation SIRS cut-off to identify severity**

**Figure S8**. **Kaplan-Meier analysis using the optimised derivation NEWS cut-off to identify severity**

**Figure S9**. **Meta-analysis to assess derivation and validation heterogeneity for hospitalisation decisions**

**Figure S10**. **Fagan nomogram for the calculation of post-test probabilities for hospitalisation decisions**

**Figure S11**. **Conventional and virtual MR-proADM guided triage in the derivation patient cohort**

**Figure S12**. **Conventional and virtual MR-proADM guided triage in the validation patient cohort**

**Supplementary Tables**

**Table S1. Initial clinical diagnoses and suspected infectious source in the derivation cohort**

| **Suspected source of infection** | **Patients**  **(*N*)** | **Hospital admission**  **(*N*, %)** | **ICU admission**  **(*N*, %)** | **28-day mortality**  ***(N*, %)** |
| --- | --- | --- | --- | --- |
| Bone and Joint | 13 | 10 (76.9%) | 0 (0.0%) | 0 (0.0%) |
| Septic Arthritis | 3 |  |  |  |
| Other | 10 |  |  |  |
| Cardiac | 6 | 5 (83.3%) | 0 (0.0%) | 1 (16.7%) |
| Endocarditis | 4 |  |  |  |
| Other | 2 |  |  |  |
| Central Nervous System | 13 | 12 (92.3%) | 4 (30.8%) | 3 (23.1%) |
| Meningitis | 8 |  |  |  |
| Other | 5 |  |  |  |
| Foreign object | 5 | 5 (100.0%) | 0 (0.0%) | 1 (20.0%) |
| Intra-abdominal | 158 | 127 (80.4%) | 2 (1.3%) | 5 (3.2%) |
| Appendicitis | 24 |  |  |  |
| Cholangitis | 27 |  |  |  |
| Cholecystitis | 12 |  |  |  |
| Colitis | 4 |  |  |  |
| Diverticulitis | 15 |  |  |  |
| Gastroenteritis | 39 |  |  |  |
| Other | 37 |  |  |  |
| Respiratory - lower | 413 | 348 (84.3%) | 13 (3.1%) | 44 (10.7%) |
| IECOPD | 33 |  |  |  |
| Pneumonia | 333 |  |  |  |
| Pulmonary consolidation | 9 |  |  |  |
| Other | 38 |  |  |  |
| Respiratory - upper | 85 | 33 (38.8%) | 2 (2.4%) | 0 (0.0%) |
| Influenza | 70 |  |  |  |
| Tonsillitis | 9 |  |  |  |
| Other | 6 |  |  |  |
| Skin and Soft Tissue | 96 | 75 (78.1%) | 1 (1.0%) | 7 (7.3%) |
| Cellulitis | 53 |  |  |  |
| Other | 43 |  |  |  |
| Surgical | 10 | 6 (60.0%) | 0 (0.0%) | 0 (0.0%) |
| Post abdominal surgery | 3 |  |  |  |
| Other | 7 |  |  |  |
| Unknown origin | 98 | 71 (72.4%) | 4 (4.1%) | 11 (11.2%) |
| Urogenital | 278 | 223 (80.2%) | 6 (2.2%) | 12 (4.3%) |
| Pyelonephritis | 34 |  |  |  |
| Urinary tract infection | 218 |  |  |  |
| Other | 26 |  |  |  |

*N:* Number; *ICU:* Intensive Care Unit; *IECOPD*: Infective Exacerbation of Chronic Obstructive Pulmonary Disease.

**Table S2**. **Validation cohort characteristics with regards to 28-day mortality**

| **Patient characteristics** | **Total patient cohort**  **(*N*=896)** | **Survivors**  **(*N*=851)** | **Non-Survivors**  **(*N*=45)** | **p-value** |
| --- | --- | --- | --- | --- |
| **Demographics** | | | | |
| Age (years) (mean, SD) | 58.8 (21.0) | 57.8 (20.9) | 77.3 (10.8) | <0.001 |
| Male gender (*N*, %) | 473 (52.8%) | 444 (52.2%) | 29 (64.4%) | 0.110 |
| **Disposition and mortality** | | | | |
| Hospital admission (*N*, %) | 567 (76.2%) | 524 (61.6%) | 43 (97.7%) | <0.001 |
| ICU admission (*N*, %) | 49 (5.5%) | 39 (4.6%) | 10 (22.2%) | <0.001 |
| 28-day mortality (*N*, %) | 45 (5.0%) |  | | |
| Hospital mortality (*N*, %) | 38 (4.2%) |  | | |
| Hospital length of stay (days)(median, Q1-Q3) | 2 [0 - 6] | 2 [0 - 6] | 0 [0 - 0] | <0.001 |
| **Presenting symptoms** | | | | |
| Blood loss (*N*, %) | 8 (0.9%) | 8 (0.9%) | 0 (0.0%) | 0.514 |
| Diarrhoea, vomiting, dysuria (*N*, %) | 71 (7.9%) | 70 (8.2%) | 1 (2.2%) | 0.146 |
| Fever (*N*, %) | 246 (27.5%) | 240 (28.2%) | 6 (13.3%) | 0.029 |
| General worsening (*N*, %) | 144 (16.1%) | 137 (16.1%) | 7 (15.6%) | 0.923 |
| Neurological symptoms (*N*, %) | 38 (4.2%) | 35 (4.1%) | 3 (6.7%) | 0.407 |
| Pain (other) (*N*, %) | 137 (15.3%) | 133 (15.6%) | 4 (8.9%) | 0.221 |
| Respiratory symptoms (*N*, %) | 155 (17.3%) | 134 (15.7%) | 21 (46.7%) | <0.001 |
| Thoracic pain (*N*, %) | 25 (27.9%) | 23 (2.7%) | 2 (4.4%) | 0.489 |
| Other (*N*, %) | 72 (8.0%) | 71 (8.3%) | 1 (2.2%) | 0.141 |
| **Infectious source** | | | | |
| Central Nervous System (*N*, %) | 42 (5.6%) | 39 (5.4%) | 3 (7.7%) | 0.249 |
| Fever of unknown origin (*N*, %) | 139 (18.4%) | 135 (18.9%) | 4 (10.3%) | 0.126 |
| Intra-abdominal (*N*, %) | 79 (10.5%) | 78 (10.9%) | 1 (2.6%) | 0.078 |
| Respiratory (*N*, %) | 258 (34.2%) | 233 (32.5%) | 25 (64.1%) | <0.001 |
| Skin and Soft Tissue (*N*, %) | 61 (8.1%) | 61 (8.5%) | 0 (0.0%) | 0.163 |
| Urogenital (*N*, %) | 164 (21.7%) | 158 (22.1%) | 6 (15.4%) | 0.414 |
| Other (*N*, %) | 53 (7.0%) | 51 (7.1%) | 2 (5.1%) | 0.556 |
| **Comorbidities** | | | | |
| COPD (*N*, %) | 52 (5.8%) | 42 (4.9%) | 10 (22.2%) | <0.001 |
| Dementia (*N*, %) | 44 (4.9%) | 37 (4.3%) | 7 (15.6%) | <0.001 |
| Hypertension (*N*, %) | 321 (35.8%) | 300 (35.3%) | 21 (46.7%) | 0.120 |
| Coronary arterial disease (*N*, %) | 64 (7.1%) | 55 (6.5%) | 9 (20.0%) | <0.001 |
| Stroke (*N*, %) | 26 (2.9%) | 23 (2.7%) | 3 (6.7%) | 0.123 |
| Substance abuse (*N*, %) | 40 (4.5%) | 38 (4.5%) | 2 (4.4%) | 0.995 |
| Coronary heart failure (*N*, %) | 51 (5.7%) | 44 (5.2%) | 7 (15.6%) | 0.003 |
| Diabetes (*N*, %) | 142 (15.8%) | 136 (16.0%) | 6 (13.3%) | 0.636 |
| Renal disease (*N*, %) | 169 (18.9%) | 146 (17.2%) | 23 (51.1%) | <0.001 |
| Malignancy (*N*, %) | 186 (20.8%) | 171 (20.1%) | 15 (33.3%) | 0.033 |
| **Biomarker concentrations** | | | | |
| MR-proADM (nmol/L) (median, Q1-Q3) | 1.03 [0.68 - 1.78] | 1.00 [0.67 - 1.65] | 3.99 [2.13 - 5.41] | <0.001 |
| PCT (ng/mL) (median, Q1-Q3) | 0.14 [0.08 - 0.48] | 0.12 [0.07 - 0.44] | 0.80 [0.21 - 6.77] | <0.001 |
| CRP (mg/L) (median, Q1-Q3) | 56 [15 - 142] | 52 [14 - 137] | 134 [80 - 221] | <0.001 |

Values expressed in percentages (%) indicate either the proportion of the total patient cohort, surviving or non-surviving patients at 28 days for each variable, where applicable. Data are presented as mean (standard deviation, SD) or median [first quartile (Q1) - third quartile (Q3)] where appropriate. The chi-square (χ^2^) test was used to determine significance between surviving and non-surviving patients for categorical variables, Student’s t-test for the variable of age, and Mann-Whitney U test for hospitalisation duration and biomarker variables. *COPD*: Chronic Obstructive Pulmonary Disease; *CRP*: C-reactive protein; *ICU*: Intensive Care Unit; *MR-proADM*: Mid-regional proadrenomedullin; *N*: Number; *PCT*: Procalcitonin.

**Table S3**. **Sepsis-2 and sepsis-3 classification in the derivation cohort**

| **Sepsis-2**  **(1=Yes)** | **Sepsis-3**  **(1=Yes)** | **Patient subgroup**  **(*N*, %)** | **Hospital admission**  **(*N*, %)** | **ICU**  **admission**  **(*N*, %)** | **28-day mortality**  **(*N*, %)** |
| --- | --- | --- | --- | --- | --- |
| 0 | 0 | 193 (16.4%) | 111 (57.5%) | 0 (0.0%) | 3 (1.6%) |
| 1 | 0 | 401 (34.1%) | 280 (69.8%) | 3 (0.7%) | 9 (2.2%) |
| 0 | 1 | 128 (10.9%) | 111 (86.7%) | 4 (3.1%) | 6 (4.7%) |
| 1 | 1 | 453 (38.6%) | 413 (91.2%) | 25 (5.5%) | 66 (14.6%) |

Percentage of each patient subgroup is reported as the proportion of the total derivation patient cohort. Hospitalisation, ICU admission and 28-day mortality percentages are subsequently reported as the proportion of each variable within each patient subgroup. *N*: Number; *ICU*: Intensive Care Unit.

**Table S4**. **Application of the 28-day mortality derivation MR-proADM cut-off in the validation cohort**

|  | **Derivation cohort**  **(*N*=1175; 7.1% mortality)** | **Validation cohort**  **(*N*=896; 5.0% mortality)** |
| --- | --- | --- |
| Sensitivity | 0.92 [0.84 - 0.96] | 0.92 [0.80 - 0.98] |
| Specificity | 0.74 [0.71 - 0.76] | 0.72 [0.69 - 0.75] |
| PPV | 0.21 [0.17 - 0.26] | 0.16 [0.14 - 0.17] |
| NPV | 0.99 [0.98 - 1.00] | 0.99 [0.98 - 1.00] |
| LR+ | 3.47 [3.08 - 3.90] | 3.25 [2.84 - 3.73] |
| LR- | 0.11 [0.06 - 0.23] | 0.12 [0.05 - 0.30] |
| Disease prevalence (%) | 7.15 [5.74 - 8.77] | 5.36 [3.98 - 7.04] |
| Diagnostic Odds Ratio | 30.67 [13.98 - 67.27] | 28.03 [9.96 - 78.86] |

An optimised derivation MR-proADM cut-off of 1.54 nmol/L was applied to the validation cohort. *LR-*: Negative likelihood ratio; *LR+*: Positive likelihood ratio; *N*: Number; *NPV*: Negative predictive value; *PPV*: Positive predictive value.

**Table S5**. **Pooled derivation and validation cohorts for 28-day mortality prediction using MR-proADM**

|  | **Derivation cohort**  **(*N*=1175; 7.1% mortality)** | **Validation cohort**  **(*N*=896; 5.0% mortality)** | **Pooled cohort**  **(*N*=2071; 6.2% mortality)** |
| --- | --- | --- | --- |
| Cut-off (nmol/L) | 1.54 | 1.63 | 1.54 |
| Sensitivity | 0.92 [0.84 - 0.96] | 0.92 [0.80 - 0.97] | 0.92 [0.86 - 0.96] |
| Specificity | 0.74 [0.71 - 0.76] | 0.75 [0.72 - 0.78] | 0.73 [0.71 - 0.75] |
| PPV | 0.21 [0.17 - 0.26] | 0.17 [0.13 - 0.22] | 0.19 [0.17 - 0.20] |
| NPV | 0.99 [0.98 - 1.00] | 0.99 [0.98 - 1.00] | 0.99 [0.99 - 1.00] |
| LR+ | 3.47 [3.08 - 3.90] | 3.63 [3.15 - 4.19] | 3.37 [3.08 - 3.68] |
| LR- | 0.11 [0.06 - 0.23] | 0.11 [0.04 - 0.29] | 0.11 [0.06 - 0.20] |
| Disease prevalence (%) | 7.15 [5.74 - 8.77] | 5.36 [3.98 - 7.04] | 6.37 [5.36 - 7.51] |

*LR-*: Negative likelihood ratio; *LR+*: Positive likelihood ratio; *N*: Number; *NPV*: Negative predictive value; *PPV*: Positive predictive value.

**Table S6**. **Cox regression analysis between high and low severity populations for biomarkers and scores in the derivation and validation cohorts, using optimised or pre-established cut-offs**

|  | | | Number of patients and mortality rate below cut-off | | Number of patients and mortality rate above cut-off | |  |
| --- | --- | --- | --- | --- | --- | --- | --- |
|  | Biomarker or clinical score | Cut-off | Patients  (*N*) | Mortality rate | Patients  (*N*) | Mortality rate | HR [95% CI] |
| Derivation cohort | MR-proADM (nmol/L)† | 1.54 | 810 | 0.9% | 365 | 21.1% | 27.2 [12.6 - 59.0] |
|  | PCT (ng/mL)† | 0.21 | 633 | 3.0% | 530 | 12.3% | 4.1 [2.5 - 6.9] |
|  | PCT (ng/mL)* | 0.25 | 665 | 3.2% | 498 | 12.7% | 4.2 [2.6 - 6.9] |
|  | Lactate (mmol/L)* | 2.0 | 487 | 4.7% | 257 | 14.0% | 3.2 [1.9 - 5.4] |
|  | Lactate (mmol/L)† | 2.2 | 526 | 4.9% | 218 | 15.1% | 3.3 [2.0 - 5.5] |
|  | CRP (mg/L)† | 67 | 717 | 3.9% | 450 | 12.2% | 3.3 [2.1 - 5.1] |
|  | SOFA (points)* | 2 | 585 | 1.9% | 590 | 12.3% | 7.0 [3.7 - 13.1] |
|  | SOFA (points)† | 3 | 822 | 2.7% | 353 | 17.6% | 7.2 [4.4 - 11.7] |
|  | qSOFA (points)† | 1 | 624 | 2.2% | 551 | 12.7% | 6.0 [3.4 - 10.6] |
|  | qSOFA (points)* | 2 | 1059 | 5.0% | 116 | 26.7% | 6.2 [4.0 - 9.7] |
|  | NEWS (points)* | 4 | 439 | 3.2% | 618 | 9.5% | 3.4 [1.9 - 5.4] |
|  | NEWS (points)† | 7 | 755 | 4.4% | 420 | 12.1% | 3.9 [2.5 - 6.1] |
|  | CRB-65 (points)** | 2 | 874 | 3.5% | 301 | 17.6% | 5.4 [3.5 - 8.4] |
| Validation cohort | MR-proADM (nmol/L)† | 1.54 | 612 | 0.7% | 284 | 14.4% | 25.6 [9.2 - 71.3] |
|  | PCT (ng/mL)† | 0.21 | 537 | 2.2% | 347 | 10.4% | 4.5 [2.5 - 8.0] |
|  | PCT (ng/mL)* | 0.25 | 571 | 2.5% | 313 | 10.9% | 4.7 [2.7 - 8.1] |
|  | CRP (mg/L)† | 67 | 413 | 2.4% | 367 | 9.3% | 3.9 [1.8 - 8.2] |

* denotes the use of a pre-established cut-off, † donates use of the optimised derivation cut-off, and ** indicates that both the pre-established and optimised derivation cut-offs were identical. *CI*: Confidence interval; *CRB-65*: Severity score for community-acquired pneumonia; *CRP*: C-reactive protein; *HR*: Hazard ratio; *MR-proADM*: Mid-regional proadrenomedullin; *N*: Number; *NEWS*: National Early Warning Score; *PCT*: Procalcitonin; *qSOFA*: quick Sequential Organ Failure Assessment; *SOFA*: Sequential Organ Failure Assessment.

**Table S7**. **Uncomplicated infection and disease progression events in the patient subgroup with biomarker or score values below their respective 28-day mortality cut-offs**

| **Biomarker or clinical score** | **Selected**  **cut-offs** | **Patients**  **(*N*)** | **Uncomplicated infections**  ***N* (%)** | **Disease progression**  ***N* (%)** |
| --- | --- | --- | --- | --- |
| PCT | <0.25 ng/mL | 665 | 594 (89.3%) | 71 (10.7%) |
| Lactate | <2.0 mmol/L | 487 | 397 (81.5%) | 90 (18.5%) |
| CRP | <67 mg/L | 717 | 628 (87.6%) | 89 (12.4%) |
| SOFA | <2 points | 585 | 536 (91.6%) | 49 (8.4%) |
| qSOFA | <2 points | 1059 | 885 (83.6%) | 174 (16.4%) |
| NEWS | <4 points | 439 | 392 (89.3%) | 47 (10.7%) |
| CRB-65 | <2 points | 874 | 768 (87.9%) | 106 (12.1%) |

*CRB-65*: Severity score for community-acquired pneumonia; *CRP*: C-reactive protein; *N*: Number; *NEWS*: National Early Warning Score; *PCT*: Procalcitonin; *qSOFA*: quick Sequential Organ Failure Assessment; *SOFA*: Sequential Organ Failure Assessment.

**Table S8**. **Patient subgroups with MR-proADM concentrations <1.54 nmol/L, combined with low biomarker or score values, showing enrichment for uncomplicated infections**

| **Biomarker or clinical score** | **Selected**  **cut-offs** | **Population size**  **(*N*)** | **Mortality rate**  ***N* (%)** | **ICU admission rate**  ***N* (%)** | **Length of hospitalisation**  **(days)** | **Uncomplicated infections**  ***N* (%)** | **Disease progression**  ***N* (%)** |
| --- | --- | --- | --- | --- | --- | --- | --- |
| PCT | <0.25 ng/mL | 571 | 6 (1.1%) | 6 (1.1%) | 2 [0 - 6] | 559 (97.9%) | 12 (2.1%) |
| Lactate | <2.0 mmol/L | 341 | 3 (0.9%) | 5 (1.5%) | 2 [0 - 7] | 334 (97.9%) | 7 (2.1%) |
| CRP | <67 mg/L | 557 | 2 (0.4%) | 5 (0.9%) | 2 [0 - 5] | 550 (98.7%) | 7 (1.3%) |
| SOFA | <2 points | 505 | 1 (0.2%) | 0 (0.0%) | 1 [0 - 5] | 504 (99.8%) | 1 (0.2%) |
| qSOFA | <2 points | 774 | 4 (0.5%) | 8 (1.0%) | 2 [0 - 6] | 764 (98.7%) | 10 (1.3%) |
| NEWS | <4 points | 357 | 0 (0.0%) | 1 (0.3%) | 1 [0 - 4] | 356 (99.7%) | 1 (0.3%) |
| CRB-65 | <2 points | 690 | 0 (0.0%) | 7 (1.0%) | 2 [0 - 6] | 683 (99.0%) | 7 (1.0%) |

*CRB-65*: Severity score for community-acquired pneumonia; *CRP*: C-reactive protein; *ICU*: Intensive Care Unit; *N*: Number; *NEWS*: National Early Warning Score; *PCT*: Procalcitonin; *qSOFA*: quick Sequential Organ Failure Assessment; *SOFA*: Sequential Organ Failure Assessment.

**Table S9**. **Patient subgroups with MR-proADM concentrations ≥1.54 nmol/L, combined with low biomarker or score values, showing enrichment for disease progression events**

| **Biomarker or clinical score** | **Selected**  **cut-offs** | **Population size**  **(*N*)** | **Mortality rate**  ***N* (%)** | **ICU admission rate**  ***N* (%)** | **Length of hospitalisation**  **(days)** | **Uncomplicated infections**  ***N* (%)** | **Disease progression**  ***N* (%)** |
| --- | --- | --- | --- | --- | --- | --- | --- |
| PCT | <0.25 ng/mL | 94 | 15 (16.0%) | 4 (4.3%) | 7 [4 - 16.5] | 35 (37.2%) | 59 (62.8%) |
| Lactate | <2.0 mmol/L | 146 | 20 (13.7%) | 5 (3.4%) | 8 [4.25 - 14] | 63 (43.2%) | 83 (56.8%) |
| CRP | <67 mg/L | 160 | 26 (16.3%) | 7 (4.4%) | 7 [4 - 14] | 78 (48.8%) | 82 (51.3%) |
| SOFA | <2 points | 80 | 10 (12.5%) | 2 (2.5%) | 9 [4 - 18] | 32 (40.0%) | 48 (60.0%) |
| qSOFA | <2 points | 285 | 49 (17.2%) | 16 (5.6%) | 7 [4 - 15.25] | 121 (42.5%) | 164 (57.5%) |
| NEWS | <4 points | 82 | 14 (17.1%) | 4 (4.9%) | 10 [5 - 19.5] | 35 (42.7%) | 47 (57.3%) |
| CRB-65 | <2 points | 184 | 31 (16.8%) | 12 (6.5%) | 7 [4 - 16] | 84 (45.7%) | 100 (54.3%) |

*CRB-65*: Severity score for community-acquired pneumonia; *CRP*: C-reactive protein; *ICU*: Intensive Care Unit; *N*: Number; *NEWS*: National Early Warning Score; *PCT*: Procalcitonin; *qSOFA*: quick Sequential Organ Failure Assessment; *SOFA*: Sequential Organ Failure Assessment.

**Table S10**. **Tests for significance in hospitalisation duration, 28-day mortality and ICU admission rates between subgroups enriched for uncomplicated infections or disease progression**

| Uncomplicated infection enriched  subgroup | Disease progression enriched subgroup | HR [95% CI] for 28-day mortality (p-value) | p-value for ICU admission | p-value for length of hospitalisation |
| --- | --- | --- | --- | --- |
| Low PCT + Low MR-proADM | Low PCT + High MR-proADM | 16.4 [7.0 - 38.7] (*p*<0.001) | 0.018 | <0.001 |
| Low Lactate + Low MR-proADM | Low Lactate + High MR-proADM | 10.7 [4.4 - 27.0] (*p*<0.001) | 0.163 | <0.001 |
| Low CRP + Low MR-proADM | Low CRP + High MR-proADM | 28.8 [11.6 - 51.3] (*p*<0.001) | 0.003 | <0.001 |
| Low SOFA + Low MR-proADM | Low SOFA + High MR-proADM | 45.5 [10.0 - 207.6] (*p*<0.001) | <0.001 | <0.001 |
| Low qSOFA + Low MR-proADM | Low qSOFA + High MR-proADM | 23.4 [11.1 - 49.3] (*p*<0.001) | <0.001 | <0.001 |
| Low NEWS + Low MR-proADM | Low NEWS + High MR-proADM | 32.6 [9.4 - 113.6] (*p*<0.001) | <0.001 | <0.001 |
| Low CRB-65 + Low MR-proADM | Low CRB-65 + High MR-proADM | 38.9 [10.1 - 53.2] (p<0.001) | <0.001 | <0.001 |

*CI*: Confidence Interval; *CRB-65*: Severity score for community-acquired pneumonia; *CRP*: C-reactive protein; *HR*: Hazard Ratio; *ICU*: Intensive Care Unit; *MR-proADM*: Mid-regional proadrenomedullin; *NEWS*: National Early Warning Score; *PCT*: Procalcitonin; *qSOFA*: quick Sequential Organ Failure Assessment; *SOFA*: Sequential Organ Failure Assessment. Low vs. High subgroups based on the following cut-offs for each biomarker or score: MR-proADM cut-off: <1.54 nmol/L; PCT cut-off: <0.25 ng/mL; Lactate cut-off: <2.0 mmol/L; CRP cut-off: <67 mg/L; SOFA cut-off: <2 points; qSOFA cut-off: <2 points; NEWS cut-off: <4 points; CRB-65 cut-off: <2 points.

**Table S11**. **Derivation and validation univariate logistic regression for hospitalisation decisions**

|  | **Biomarker or clinical score** | **Patients**  **(*N*)** | **Events**  **(*N*)** | **LR χ²** | **D.F.** | **p-value** | **C-index** | **OR**  **IQR [95% CI]** |
| --- | --- | --- | --- | --- | --- | --- | --- | --- |
| Derivation cohort | MR-proADM | 1175 | 918 | 259.6 | 1 | <0.001 | 0.816 | 7.40 [5.46 - 10.03] |
|  | PCT | 1163 | 906 | 124.1 | 1 | <0.001 | 0.719 | 3.29 [2.58 - 4.20] |
|  | Lactate | 743 | 618 | 8.2 | 1 | <0.001 | 0.602 | 1.42 [1.11 - 1.80] |
|  | CRP | 1167 | 911 | 20.5 | 1 | <0.001 | 0.601 | 1.60 [1.31 - 1.96] |
|  | SOFA | 1175 | 918 | 165.0 | 1 | <0.001 | 0.746 | 8.23 [5.52 - 12.28] |
|  | qSOFA | 1175 | 918 | 50.4 | 1 | <0.001 | 0.623 | 2.29 [1.78 - 2.93] |
|  | NEWS | 1055 | 827 | 86.2 | 1 | <0.001 | 0.688 | 3.27 [2.48 - 4.31] |
|  | CRB-65 | 1175 | 918 | 134.2 | 1 | <0.001 | 0.712 | 3.07 [2.47 - 3.81] |
| Validation cohort | MR-proADM | 744 | 576 | 211.7 | 1 | <0.001 | 0.839 | 12.97 [8.16 - 20.60] |
|  | PCT | 733 | 572 | 85.1 | 1 | <0.001 | 0.749 | 3.72 [2.58 - 5.35] |
|  | CRP | 629 | 500 | 70.0 | 1 | <0.001 | 0.761 | 2.34 [1.89 - 2.89] |

*CI*: Confidence Interval; *CRB-65*: Severity score for community-acquired pneumonia; *CRP*: C-reactive protein; *DF*: Degrees of Freedom; *IQR*: Interquartile range; *LR*: Likelihood ratio; *MR-proADM*: Mid-regional proadrenomedullin; *N*: Number; *NEWS*: National Early Warning Score; *OR*: Odds Ratio; *PCT*: Procalcitonin; *qSOFA*: quick Sequential Organ Failure Assessment; *SOFA*: Sequential Organ Failure Assessment.

**Table S12**. **Derivation and validation multivariate logistic regression for hospitalisation decisions**

|  | **Biomarker or clinical score** | **Patients**  **(*N*)** | **Events**  **(*N*)** | **LR χ²** | **D.F.** | **p-value** | **C-index** | **OR**  **IQR [95% CI]** |
| --- | --- | --- | --- | --- | --- | --- | --- | --- |
| Derivation cohort | MR-proADM | 1175 | 918 | 277.5 | 6 | <0.001 | 0.821 | 5.14 [3.68 - 7.17] |
|  | PCT | 1163 | 906 | 234.8 | 6 | <0.001 | 0.799 | 2.81 [2.19 - 3.61] |
|  | Lactate | 743 | 618 | 92.6 | 6 | <0.001 | 0.754 | 1.18 [0.91 - 1.53] |
|  | CRP | 1167 | 911 | 168.5 | 6 | <0.001 | 0.755 | 1.61 [1.29 - 2.01] |
|  | SOFA | 1175 | 918 | 228.5 | 6 | <0.001 | 0.787 | 4.80 [3.20 - 7.22] |
|  | qSOFA | 1175 | 918 | 168.1 | 6 | <0.001 | 0.751 | 1.71 [1.31 - 2.23] |
|  | NEWS | 1055 | 827 | 184.3 | 6 | <0.001 | 0.780 | 2.66 [1.98 - 3.57] |
|  | CRB-65 | 1175 | 918 | 172.6 | 6 | <0.001 | 0.755 | 1.80 [1.39 - 2.34] |
| Validation cohort | MR-proADM | 744 | 576 | 262.0 | 6 | <0.001 | 0.867 | 10.01 [5.67 - 17.65] |
|  | PCT | 733 | 572 | 208.9 | 6 | <0.001 | 0.842 | 2.93 [2.02 - 4.25] |
|  | CRP | 629 | 500 | 169.1 | 6 | <0.001 | 0.840 | 1.93 [1.53 - 2.45] |

Age, cardiovascular, neurological, renal and malignancy comorbidities were used as adjusting variables within the multivariate derivation cohort model, and subsequently applied to the validation cohort. *CI*: Confidence Interval; *CRB-65*: Severity score for community-acquired pneumonia; *CRP*: C-reactive protein; *DF*: Degrees of Freedom; *IQR*: Interquartile range; *LR*: Likelihood ratio; *MR-proADM*: Mid-regional proadrenomedullin; *N*: Number; *NEWS*: National Early Warning Score; *OR*: Odds Ratio; *PCT*: Procalcitonin; *qSOFA*: quick Sequential Organ Failure Assessment; *SOFA*: Sequential Organ Failure Assessment.

**Table S13**. **Application of the optimised derivation MR-proADM cut-off for hospitalisation in the derivation and validation cohort**

|  | **Derivation cohort**  **(*N*=1175; 77.9% hospitalisation)** | **Validation cohort**  **(*N*=896*; 76.2% hospitalisation)** |
| --- | --- | --- |
| Sensitivity | 0.75 [0.72 - 0.78] | 0.73 [0.70 - 0.77] |
| Specificity | 0.80 [0.74 - 0.84] | 0.78 [0.71 - 0.84] |
| PPV | 0.93 [0.91 - 0.95] | 0.92 [0.90 - 0.94] |
| NPV | 0.48 [0.43 - 0.52] | 0.46 [0.42 - 0.50] |
| LR+ | 3.69 [2.90 - 4.71] | 3.33 [2.50 - 4.45] |
| LR- | 0.31 [0.27 - 0.35] | 0.34 [0.29 - 0.40] |
| Prevalence (%) | 77.87 [75.39 - 80.22] | 77.42 [74.24 - 80.38] |
| Diagnostic Odds Ratio | 11.91 [8.50 - 16.68] | - 1. [6.50 - 14.74] |

Optimised derivation cut-off for MR-proADM was 0.87 nmol/L. * donates that no hospitalisation data was available for 152 patients from the validation cohort. *LR-*: Negative likelihood ratio; *LR+*: Positive likelihood ratio; *N*: Number; *NPV*: Negative predictive value; *PPV*: Positive predictive value.

**Table S14**. **Derivation and validation logistic regression analysis for hospitalisation using the optimised derivation cut-offs**

|  | Biomarker or clinical score | Cut-off | Logistic regression for hospitalisation decisions above vs. below cut-off  OR [95% CI] |
| --- | --- | --- | --- |
| Derivation cohort | MR-proADM | 0.87 nmol/L | 11.9 [8.5 - 16.7] |
|  | PCT | 0.18 ng/mL | 4.3 [3.1 - 5.8] |
|  | Lactate | 1.6 mmol/L | 2.1 [1.4 - 3.2] |
|  | CRP | 69 mg/L | 2.4 [1.8 - 3.4] |
|  | SOFA | 1 point | 5.5 [4.1 - 7.3] |
|  | qSOFA | 1 point | 2.6 [1.9 - 3.5] |
|  | NEWS | 4 points | 2.9 [2.2 - 4.0] |
|  | CRB-65 | 1 point | 4.6 [3.4 - 6.1] |
| Validation cohort | MR-proADM | 0.87 nmol/L | 8.4 [5.7 - 1 2.4] |
|  | PCT | 0.18 ng/mL | 3.3 [2.2 - 4.9] |
|  | CRP | 69 mg/L | 3.4 [2.1 - 4.8] |

*CRB-65*: Severity score for community-acquired pneumonia; *CRP*: C-reactive protein; *MR-proADM*: Mid-regional proadrenomedullin; *NEWS*: National Early Warning Score; *PCT*: Procalcitonin; *qSOFA*: quick Sequential Organ Failure Assessment; *SOFA*: Sequential Organ Failure Assessment.

**Table S15**. **Pooled derivation and validation cohorts for hospitalisation decisions using MR-proADM**

|  | **Derivation cohort**  **(*N*=1175; 77.9% hospitalisation)** | **Validation cohort**  **(*N*=896; 76.2% hospitalisation)** | **Pooled cohort**  **(*N*=2071*; 77.2% hospitalisation)** |
| --- | --- | --- | --- |
| Cut-off (nmol/L) | 0.87 | 0.89 | 0.89 |
| Sensitivity | 0.75 [0.72 - 0.78] | 0.73 [0.70 - 0.77] | 0.74 [0.71 - 0.76] |
| Specificity | 0.80 [0.74 - 0.84] | 0.80 [0.74 - 0.86] | 0.80 [0.76 - 0.83] |
| PPV | 0.93 [0.91 - 0.95] | 0.93 [0.90 - 0.95] | 0.93 [0.91 - 0.94] |
| NPV | 0.48 [0.43 - 0.52] | 0.47 [0.41 - 0.52] | 0.47 [0.44 - 0.49] |
| LR+ | 3.69 [2.90 - 4.71] | 3.73 [2.74 - 5.08] | 3.70 [3.06 - 4.49] |
| LR- | 0.31 [0.27 - 0.35] | 0.33 [0.29 - 0.39] | 0.33 [0.30 - 0.36] |
| Prevalence (%) | 77.87 [75.39 - 80.22] | 76.21 [72.98 - 79.23] | - 1. [75.28 - 79.09] |

* donates that no hospitalisation data was available for 152 patients from the validation cohort. *LR-*: Negative likelihood ratio; *LR+*: Positive likelihood ratio; *N*: Number; *NPV*: Negative predictive value; *PPV*: Positive predictive value.

**Supplementary Figures**

**Figure S1**. **Meta-analysis to assess derivation and validation heterogeneity for 28-day mortality**

**A**

**B**

**Figure S1**. Meta-analysis to assess combined derivation and validation hazard ratio (HR [95% CI]) and heterogeneity (*I*^2^) between cohorts, for MR-proADM in predicting 28-day mortality. Meta-analysis were made for derivation and validation univariate (Panel A) and multivariate (Panel B) Cox regression analyses. Statistical analysis was performed using the MIDAS module for STATA software, version 12.0 (Stata Corporation, College Station, TX, USA).

**Figure S2**. **Fagan nomogram for the calculation of post-test probabilities for 28-day mortality**

**B**

**A**

**Figure S2**. Fagan nomogram of the MR-proADM test for the calculation of post-test probabilities for 28-day mortality. Fixed pre-test probabilities of 5% (Panel A) and 20% (Panel B) for 28-day mortality were investigated. *LR*: Likelihood ratio; *Neg*: Negative; *Pos*: Positive. Statistical analysis was performed using the MIDAS module for STATA software, version 12.0 (Stata Corporation, College Station, TX, USA).

**Figure S3**. **Kaplan-Meier analysis using the optimised derivation lactate cut-off to identify severity**

**
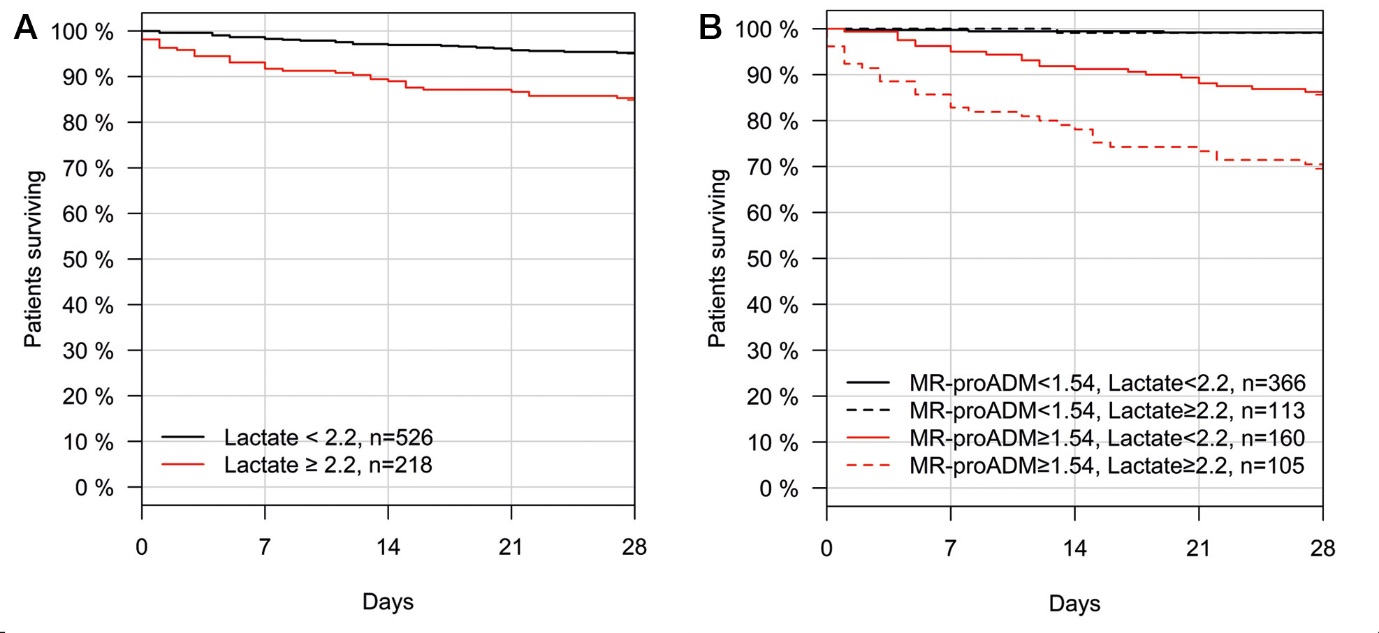
**

| **Panel B** | | |
| --- | --- | --- |
| **Reference Group 1** | **Reference Group 2** | **HR [95% CI]** |
| MR-proADM <1.54, Lactate <2.2 | MR-proADM <1.54, Lactate ≥2.2 | 0.60 [ 0.07 - 4.97] |
| MR-proADM <1.54, Lactate <2.2 | MR-proADM ≥1.54, Lactate <2.2 | 12.20 [ 4.97 - 29.97] |
| MR-proADM <1.54, Lactate <2.2 | MR-proADM ≥1.54, Lactate ≥2.2 | 30.11 [12.59 - 72.04] |
| MR-proADM <1.54, Lactate ≥2.2 | MR-proADM <1.54, Lactate <2.2 | 1.67 [0.20 - 13.88] |
| MR-proADM <1.54, Lactate ≥2.2 | MR-proADM ≥1.54, Lactate <2.2 | 20.40 [2.75 - 151.02] |
| MR-proADM <1.54, Lactate ≥2.2 | MR-proADM ≥1.54, Lactate ≥2.2 | 50.33 [6.88 - 368.34] |

**Figure S3**. Kaplan-Meier analysis within the derivation patient population using lactate at a cut-off of 2.2 mmol/L (Panel A) and in combination with MR-proADM (Panel B). *CI*: Confidence Interval; *HR*: Hazard ratio; *MR-proADM*: Mid-regional proadrenomedullin.

**Figure S4**. **Kaplan-Meier analysis using the optimised derivation PCT cut-off to identify severity**

**
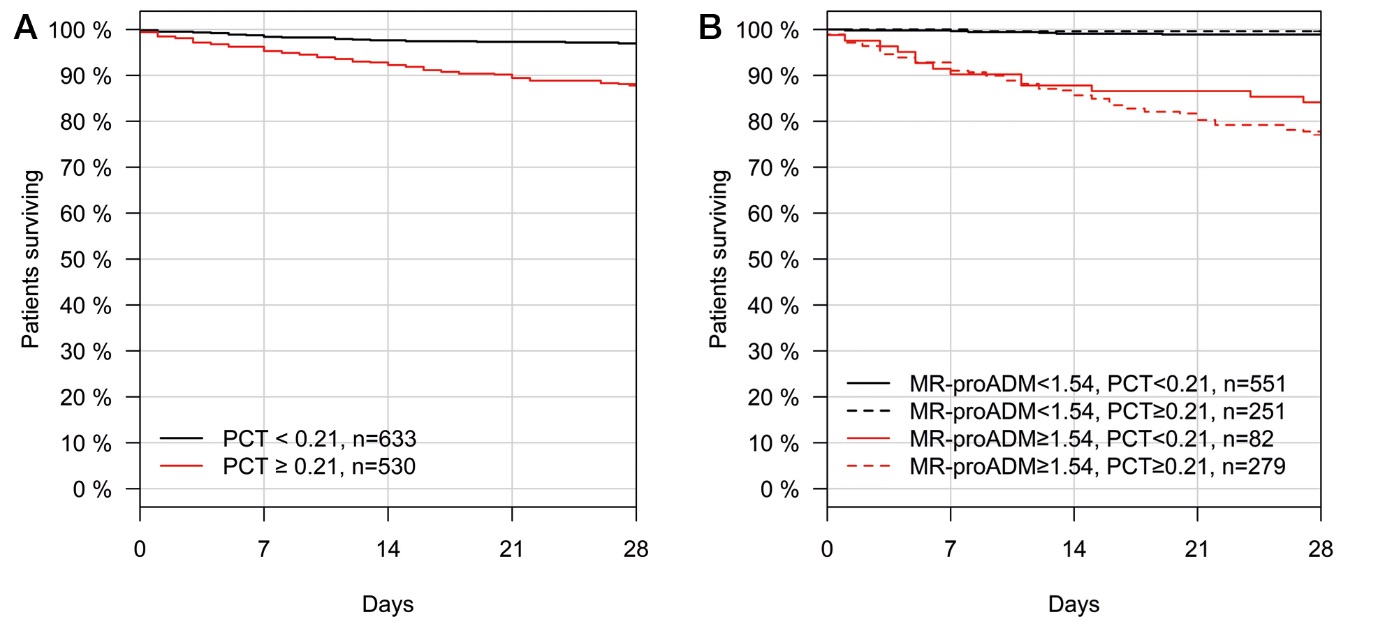
**

| **Panel B** | | |
| --- | --- | --- |
| **Reference Group 1** | **Reference Group 2** | **HR [95% CI]** |
| MR-proADM <1.54, PCT <0.21 | MR-proADM <1.54, PCT ≥0.21 | 1.44 [0.43 - 4.78] |
| MR-proADM <1.54, PCT <0.21 | MR-proADM ≥1.54, PCT <0.21 | 15.85 [6.57 - 38.24] |
| MR-proADM <1.54, PCT <0.21 | MR-proADM ≥1.54, PCT ≥0.21 | 25.19 [12.08 - 52.50] |
| MR-proADM <1.54, PCT ≥0.21 | MR-proADM <1.54, PCT <0.21 | 0.69 [0.21 - 2.31] |
| MR-proADM <1.54, PCT ≥0.21 | MR-proADM ≥1.54, PCT <0.21 | 11.00 [3.59 - 33.74] |
| MR-proADM <1.54, PCT ≥0.21 | MR-proADM ≥1.54, PCT ≥0.21 | 17.48 [6.37 - 47.99] |

**Figure S4**. Kaplan-Meier analysis within the derivation patient population using PCT at an optimised cut-off of 0.21 ng/mL (Panel A) and in combination with MR-proADM (Panel B). *CI*: Confidence Interval; *HR*: Hazard ratio; *MR-proADM*: Mid-regional proadrenomedullin; *PCT*: Procalcitonin.

**Figure S5**. **Kaplan-Meier analysis using the optimised derivation qSOFA cut-off to identify severity**

**
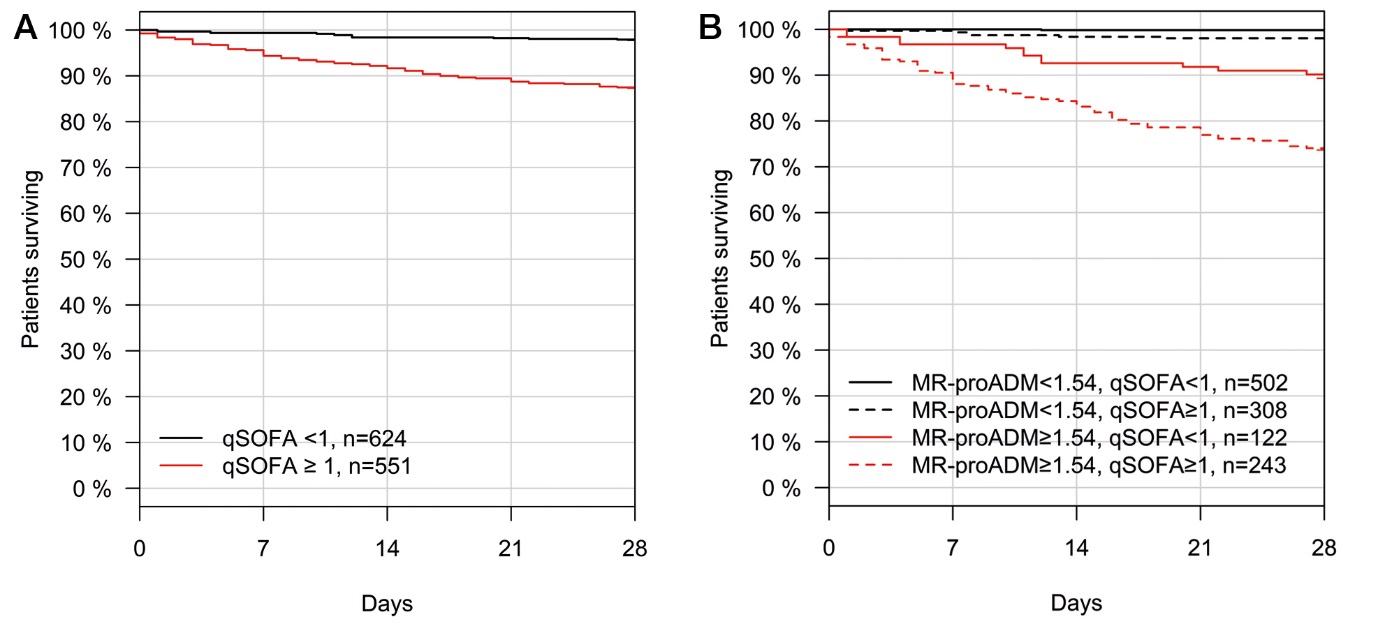
**

| **Panel B** | | |
| --- | --- | --- |
| **Reference Group 1** | **Reference Group 2** | **HR [95% CI]** |
| MR-proADM <1.54, qSOFA <1 | MR-proADM <1.54, qSOFA ≥1 | 10.61 [2.32 - 48.41] |
| MR-proADM <1.54, qSOFA <1 | MR-proADM ≥1.54, qSOFA <1 | 38.54 [8.70 - 170.78] |
| MR-proADM <1.54, qSOFA <1 | MR-proADM ≥1.54, qSOFA ≥1 | 108.74 [26.62 - 444.16] |
| MR-proADM <1.54, qSOFA ≥1 | MR-proADM <1.54, qSOFA <1 | 0.09 [0.02 - 0.43] |
| MR-proADM <1.54, qSOFA ≥1 | MR-proADM ≥1.54, qSOFA <1 | 3.63 [1.59 - 8.29] |
| MR-proADM <1.54, qSOFA ≥1 | MR-proADM ≥1.54, qSOFA ≥1 | 10.25 [5.27 - 19.96] |

**Figure S5**. Kaplan-Meier analysis within the derivation patient population using qSOFA at an optimised cut-off of 1 point (Panel A) and in combination with MR-proADM (Panel B). *CI*: Confidence Interval; *HR*: Hazard ratio; *MR-proADM*: Mid-regional proadrenomedullin; *qSOFA*: quick Sequential Organ Failure Assessment.

**Figure S6**. **Kaplan-Meier analysis using the optimised derivation SOFA cut-off to identify severity**

**
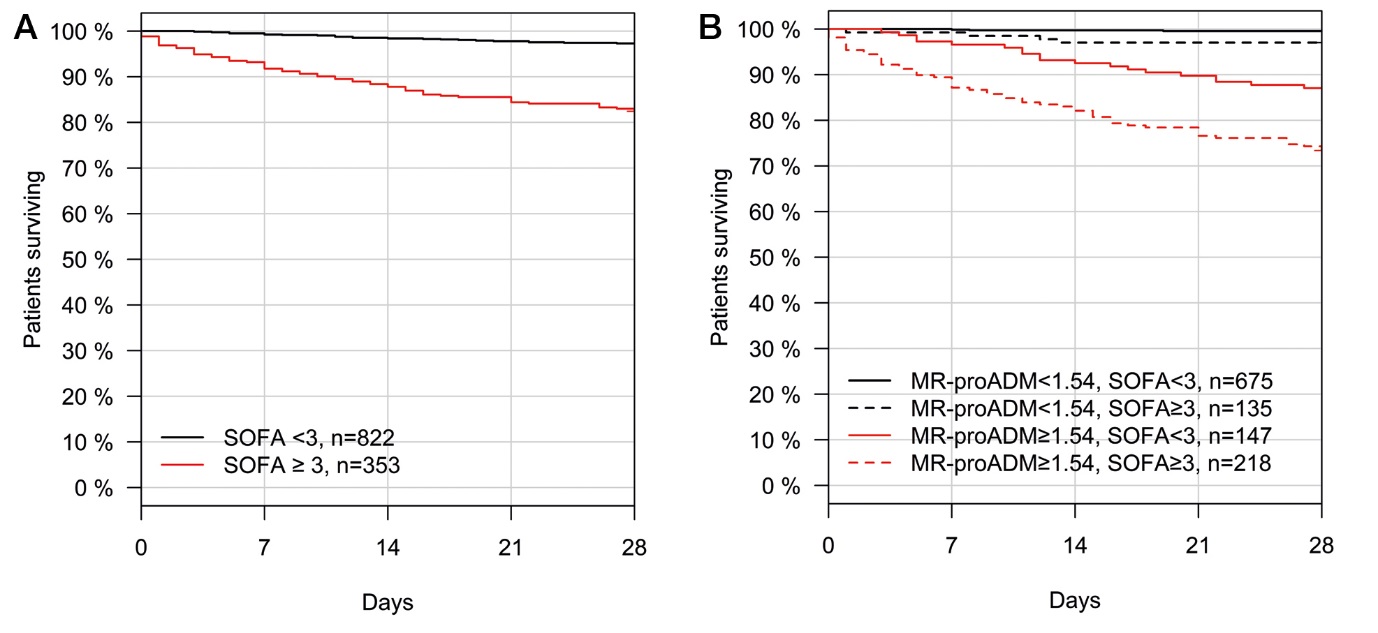
**

| **Panel B** | | |
| --- | --- | --- |
| **Reference Group 1** | **Reference Group 2** | **HR [95% CI]** |
| MR-proADM <1.54, SOFA <3 | MR-proADM <1.54, SOFA ≥3 | 6.37 [2.06 - 19.72] |
| MR-proADM <1.54, SOFA <3 | MR-proADM ≥1.54, SOFA <3 | 19.95 [7.98 - 49.91] |
| MR-proADM <1.54, SOFA <3 | MR-proADM ≥1.54, SOFA ≥3 | 46.71 [20.18 - 108.09] |
| MR-proADM <1.54, SOFA ≥3 | MR-proADM <1.54, SOFA <3 | 0.16 [0.05 - 0.49] |
| MR-proADM <1.54, SOFA ≥3 | MR-proADM ≥1.54, SOFA <3 | 3.13 [1.25 - 7.84] |
| MR-proADM <1.54, SOFA ≥3 | MR-proADM ≥1.54, SOFA ≥3 | 7.33 [3.17 - 16.97] |

**Figure S6**. Kaplan-Meier analysis within the derivation patient population using SOFA at an optimised cut-off of 3 points (Panel A) and in combination with MR-proADM (Panel B). *CI*: Confidence Interval; *HR*: Hazard ratio; *MR-proADM*: Mid-regional proadrenomedullin; *SOFA*: Sequential Organ Failure Assessment.

**Figure S7**. **Kaplan-Meier analysis using the optimised derivation SIRS cut-off to identify severity**

**
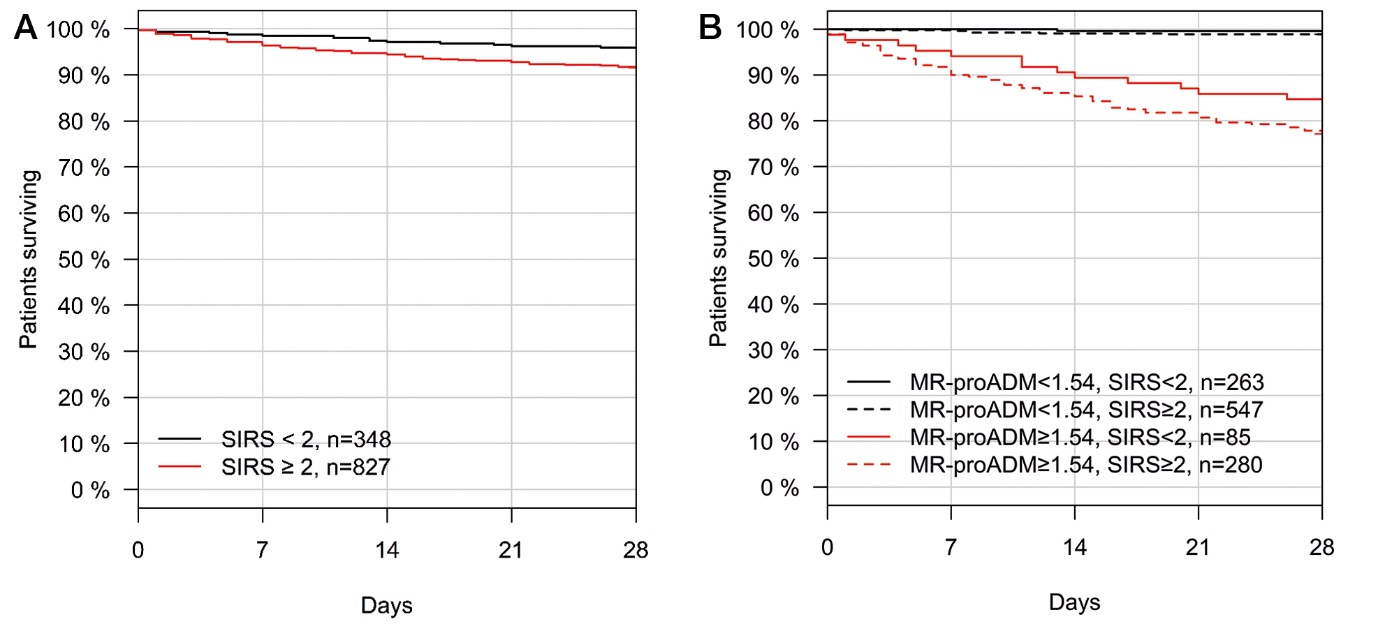
**

| **Panel B** | | |
| --- | --- | --- |
| **Reference Group 1** | **Reference Group 2** | **HR [95% CI]** |
| MR-proADM <1.54, SIRS <2 | MR-proADM <1.54, SIRS ≥2 | 2.44 [0.66 - 9.01] |
| MR-proADM <1.54, SIRS <2 | MR-proADM ≥1.54, SIRS <2 | 23.92 [6.82 - 83.94] |
| MR-proADM <1.54, SIRS <2 | MR-proADM ≥1.54, SIRS ≥2 | 41.09 [12.91 - 130.76] |
| MR-proADM <1.54, SIRS ≥2 | MR-proADM <1.54, SIRS <2 | 0.41 [0.11 - 1.51] |
| MR-proADM <1.54, SIRS ≥2 | MR-proADM ≥1.54, SIRS <2 | 9.80 [4.19 - 22.93] |
| MR-proADM <1.54, SIRS ≥2 | MR-proADM ≥1.54, SIRS ≥2 | 16.84 [8.39 - 33.82] |

**Figure S7**. Kaplan-Meier analysis within the derivation patient population using SIRS at cut-off of 2 points (Panel A) and in combination with MR-proADM (Panel B). *CI*: Confidence Interval; *HR*: Hazard ratio; *MR-proADM*: Mid-regional proadrenomedullin; *SIRS*: Systemic Inflammatory Response Syndrome.

**Figure S8**. **Kaplan-Meier analysis using the optimised derivation NEWS cut-off to identify severity**

| **Panel B** | | |
| --- | --- | --- |
| **Reference Group 1** | **Reference Group 2** | **HR [95% CI]** |
| MR-proADM <1.54, NEWS <5 | MR-proADM <1.54, NEWS ≥5 | 6.64 [1.80 - 24.51] |
| MR-proADM <1.54, NEWS <5 | MR-proADM ≥1.54, NEWS <5 | 37.15 [10.99 - 125.54] |
| MR-proADM <1.54, NEWS <5 | MR-proADM ≥1.54, NEWS ≥5 | 61.47 [19.24 - 196.41] |
| MR-proADM <1.54, NEWS ≥5 | MR-proADM <1.54, NEWS <5 | 0.15 [0.04 - 0.56] |
| MR-proADM <1.54, NEWS ≥5 | MR-proADM ≥1.54, NEWS <5 | 5.60 [2.53 - 12.37] |
| MR-proADM <1.54, NEWS ≥5 | MR-proADM ≥1.54, NEWS ≥5 | 9.26 [4.58 - 18.73] |

**
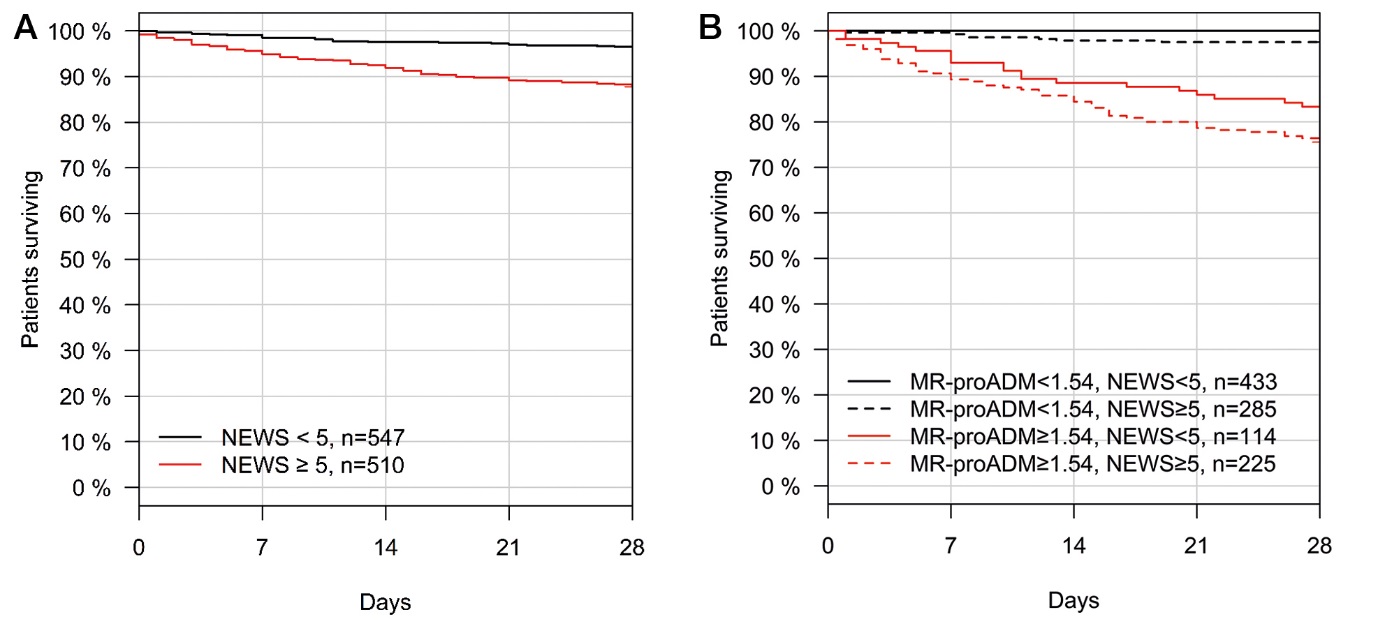
**

**Figure S8**. Kaplan-Meier analysis within the derivation patient population using NEWS at cut-off of 5 points (Panel A) and in combination with MR-proADM (Panel B). *CI*: Confidence Interval; *HR*: Hazard ratio; *MR-proADM*: Mid-regional proadrenomedullin; *NEWS*: National Early Warning Score.

**Figure S9**. **Meta-analysis to assess derivation and validation heterogeneity for hospitalisation decisions**

**B**

**A**

**Figure S9**. Meta-analysis to assess combined derivation and validation hazard ratio (HR [95% CI]) and heterogeneity (*I*^2^) between cohorts, for MR-proADM in predicting hospitalisation. Meta-analysis were made for derivation and validation univariate (Panel A) and multivariate (Panel B) Cox regression analyses. Statistical analysis was performed using the MIDAS module for STATA software, version 12.0 (Stata Corporation, College Station, TX, USA).

**Figure S10**. **Fagan nomogram for the calculation of post-test probabilities for hospitalisation decisions**

**B**

**A**

**Figure S10**. Fagan nomogram of the MR-proADM test for the calculation of post-test probabilities for hospitalisation decisions. Fixed pre-test probabilities of 5% (Panel A) and 20% (Panel B) for hospitalisation decisions were investigated. *LR*: Likelihood ratio; *Neg*: Negative; *Pos*: Positive. Statistical analysis was performed using the MIDAS module for STATA software, version 12.0 (Stata Corporation, College Station, TX, USA).

**Figure S11**. **Conventional and virtual MR-proADM guided triage in the derivation patient cohort**

**
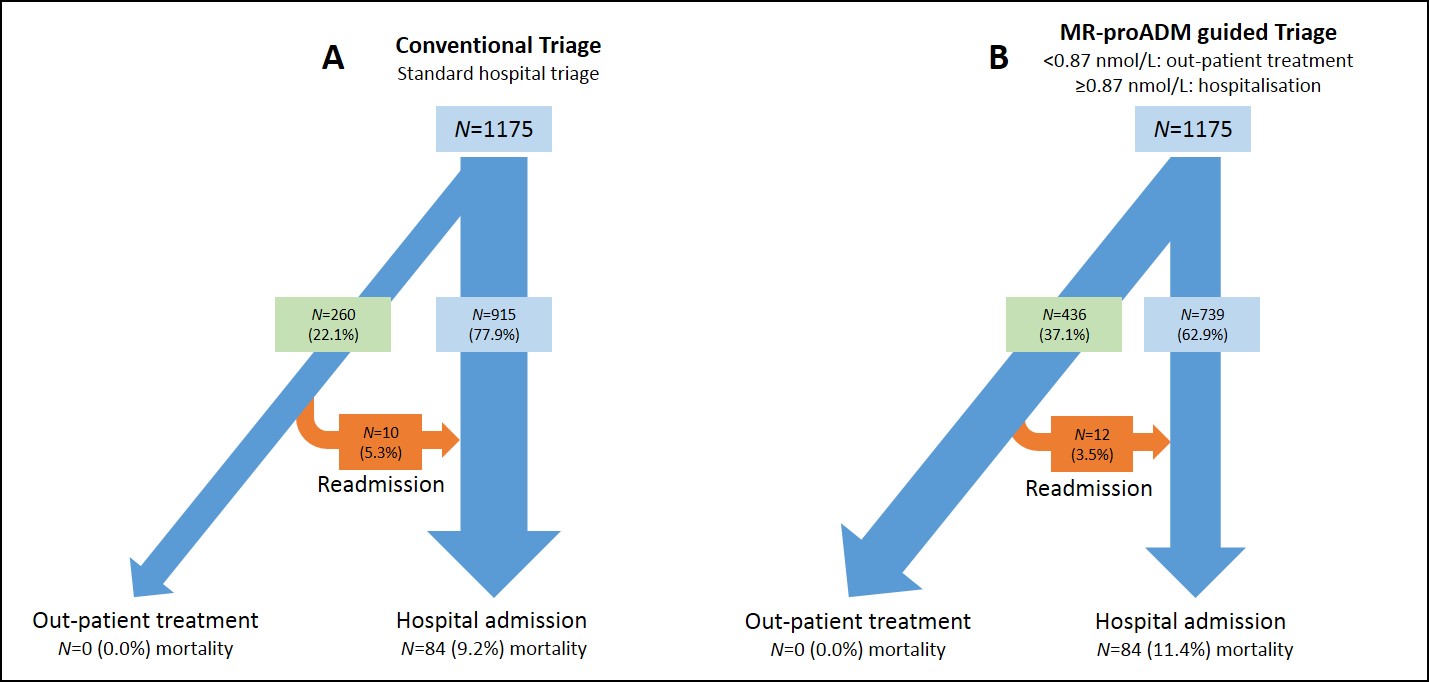
**

Patient triage decisions in the derivation study cohort, illustrating hospitalisation and out-patient treatment decisions after initial Emergency Department assessment, with corresponding mortality and re-hospitalisation rates. (Panel A): conventional “real-life” hospital triage decisions, and (Panel B): virtual MR-proADM guided triage decisions.

**Figure S12**. **Conventional and virtual MR-proADM guided triage in the validation patient cohort**

**
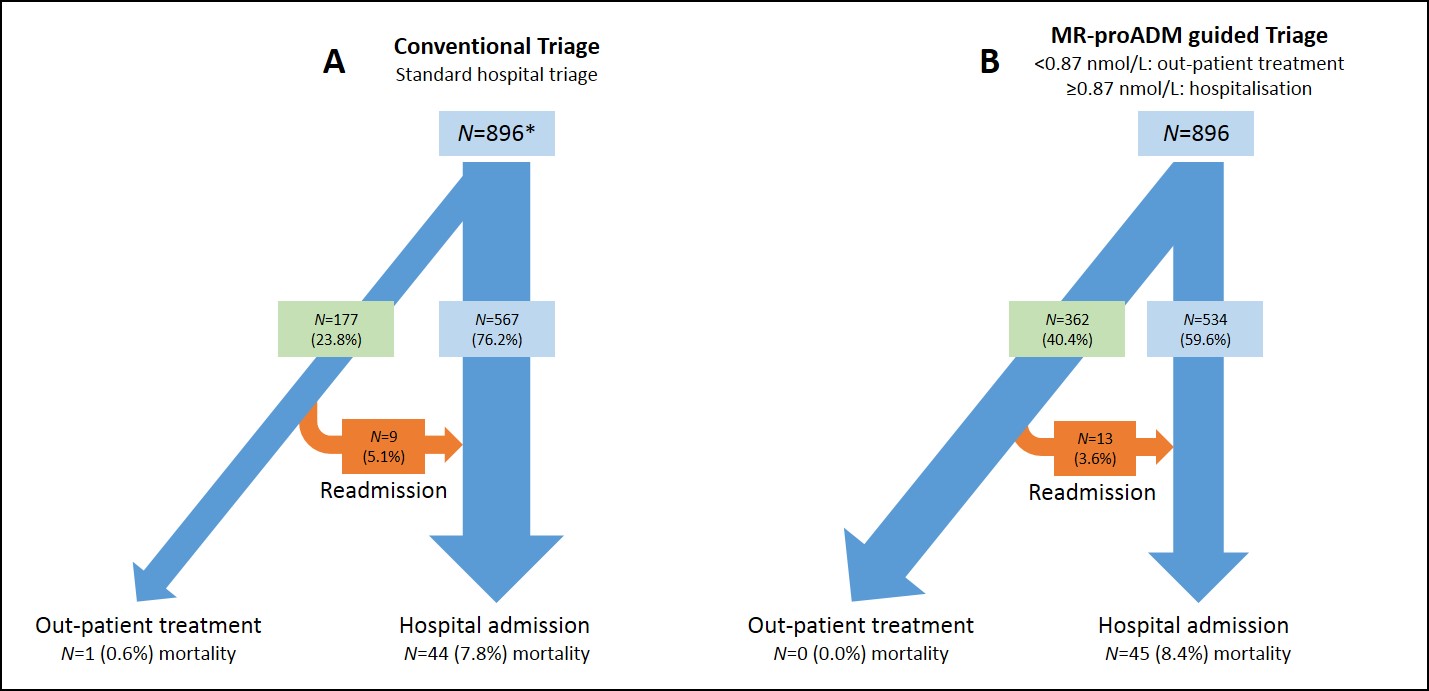
**

* donates that no hospitalisation data was available for 152 patients from the validation cohort. Patient triage decisions in the validation study cohort, illustrating hospitalisation and out-patient treatment decisions after initial Emergency Department assessment, with corresponding mortality and re-hospitalisation rates. (Panel A): conventional “real-life” hospital triage decisions, and (Panel B): virtual MR-proADM guided triage decisions.
